# Advancing whole‐brain BOLD functional MRI in humans at 10.5 T with motion‐robust 3D echo‐planar imaging, parallel transmission, and high‐density radiofrequency receive coils

**DOI:** 10.1002/mrm.70110

**Published:** 2025-10-17

**Authors:** Shuxian Qu, Jiaen Liu, Peter van Gelderen, Jacco A. de Zwart, Jeff H. Duyn, Matt Waks, Russell Lagore, Alexander Bratch, Andrea Grant, Edward Auerbach, Lance Delabarre, Alireza Sadeghi‐Tarakameh, Yigitcan Eryaman, Gregor Adriany, Kamil Ugurbil, Xiaoping Wu

**Affiliations:** ^1^ Center for Magnetic Resonance Research, Radiology, Medical School, University of Minnesota Twin Cities Minneapolis Minnesota USA; ^2^ Advanced Imaging Research Center, UT Southwestern Medical Center Dallas Texas USA; ^3^ Radiology, UT Southwestern Medical Center Dallas Texas USA; ^4^ Advanced MRI section, NINDS, NIH Bethesda Maryland USA

**Keywords:** 3D EPI, BOLD fMRI, High‐density RF coils, Parallel transmission, Resting‐state fMRI, Ultrahigh‐field MRI

## Abstract

**Purpose:**

To demonstrate the feasibility and performance of whole‐brain blood oxygen level–dependent functional MRI (fMRI) in humans at 10.5 T by combining motion‐robust three‐dimensional gradient‐echo echo‐planar imaging, parallel transmission, and high‐density radiofrequency (RF) receive coils.

**Methods:**

Resting‐state fMRI time series were collected in healthy adults at 1.58 mm and approximately 2‐s spatiotemporal resolution using a custom‐built 16‐channel transmit/80‐channel receive RF array. Individualized parallel‐transmission, spatial‐spectral RF pulses were designed to achieve uniform water‐selective excitation across the entire brain without the need for additional fat saturation. Images were reconstructed with navigator‐guided joint motion and field correction. Reconstructed images were preprocessed using fMRIPrep and postprocessed using XCP‐D pipelines. Relevant resting‐state fMRI metrics were evaluated including temporal SNR (tSNR), amplitude of low‐frequency fluctuation, and regional homogeneity. The results were compared with those obtained using uncorrected reconstruction (i.e., using same raw data but without motion or field correction).

**Results:**

Our motion‐corrected reconstruction largely improved image quality for fMRI time series, reducing motion confounds when compared with uncorrected reconstruction. The reduction in motion confounds translated into an improvement in tSNR, with tSNR averaged across all volunteers being increased by about 11%. Our motion‐corrected reconstruction also improved both amplitude of low‐frequency fluctuation and regional homogeneity in most cortical surfaces and subcortical regions.

**Conclusion:**

It is feasible to perform quality three‐dimensional whole‐brain blood oxygen level–dependent fMRI in humans at 10.5 T using a new comprehensive motion‐robust imaging method. This work paves the way for promising future applications at 10.5 T aimed at studying brain function and networks with high spatiotemporal resolution.

## 1 INTRODUCTION

Functional MRI (fMRI)^1^, ^2^, ^3^ has become an indispensable tool for investigating human‐brain function, enabling the noninvasive mapping of neural activity through the blood oxygen level–dependent (BOLD) contrast.^4^ The pursuit of higher spatiotemporal resolution in fMRI has driven the development of ultrahigh‐field (UHF) MRI systems^5^, ^6^, ^7^, ^8^ with magnetic field strengths of 7 T and beyond,^5^, ^7^ including those operating at greater than 10 T.^5^, ^9^ These UHF systems offer increased signal‐to‐noise (SNR) and contrast‐to‐noise ratios,^10^, ^11^, ^12^, ^13^, ^14^, ^15^, ^16^ which can be leveraged to achieve finer spatial detail or faster temporal sampling. However, the benefits of UHF MRI are accompanied by significant technical challenges,^17^, ^18^ including exacerbated B_0_ and B_1_ field inhomogeneities,^19^ which can compromise image quality and the reliability of functional measurements.

Great strides have been made to advance human‐brain fMRI at 7 T^20^, ^21^, ^22^, ^23^, ^24^, ^25^, ^26^, ^27^ with established methods for mitigating UHF‐related artifacts (such as RF shading artifact).^28^, ^29^, ^30^, ^31^, ^32^ However, the extension to 10.5 T, which has been demonstrated feasible and safe,^7^, ^33^ requires further innovations to handle the amplified challenges. At such extreme field strengths, issues such as subject motion and motion‐induced B_0_ field variations^34^ become even more critical. This is particularly the case when pursuing fMRI with whole‐brain coverage, necessitating advanced strategies for data acquisition and correction.

To address these challenges, we have developed a comprehensive approach for whole‐brain BOLD fMRI at 10.5 T that integrates several leading‐edge techniques. Central to our method is a motion‐robust 3D gradient echo (GRE) echo‐planar imaging (EPI) sequence combined with custom‐built RF head arrays allowing parallel transmission (pTx)^35^, ^36^ and parallel imaging.^37^, ^38^ The motion robustness is accomplished by incorporating volumetric navigators (collected in the same sequence) in image reconstruction to correct for both intravolume and intervolume rigid‐body head motion and motion‐induced B_0_ field changes. This retrospective navigator‐informed joint correction framework has been developed in our previous work,^39^ and its utility has been demonstrated in the context of high‐resolution human‐brain T_2_*‐weighted anatomic imaging at UHF.^40^, ^41^, ^42^, ^43^, ^44^, ^45^ To overcome transmit B_1_ (B_1_^+^) inhomogeneities, pTx spatial‐spectral RF pulses^46^ are designed for achieving uniform water‐selective excitation across the entire brain. Signal detection is enhanced using high‐density RF receive coils,^11^, ^16^ which provide superior SNR and support high acceleration in parallel imaging.

In this paper, we describe the implementation and evaluation of this integrated approach for whole‐brain GRE‐BOLD fMRI at 10.5 T in healthy humans. We acquire resting‐state fMRI (rs‐fMRI) data using a custom‐built RF head array^16^ optimized for 16‐channel transmission and 80‐channel reception (16Tx80Rx). We assess the performance of our method with respect to image quality, artifact mitigation, and sensitivity to functional activation. Preliminary results are reported in the form of a conference abstract.^47^ Our findings demonstrate that, by synergistically combining these advanced technologies, it is feasible to obtain quality whole‐brain fMRI data at the UHF of 10.5 T. This work not only pushes the boundaries of current fMRI technology but also paves the way for future studies that can exploit the unique advantages of UHF MRI to uncover new insights into brain function and dysfunction. The development of robust techniques for fMRI at 10.5 T is particularly timely, as it aligns with the growing interest in UHF MRI for both research and clinical applications, offering unprecedented opportunities to study the human brain in greater detail.

## 2 METHODS

### 2.1 MRI experiments

We performed human experiments on a Siemens Magnetom 10.5T MR scanner (Siemens Healthineers, Erlangen, Germany) retrofitted with 16‐channel RF transmission and 128‐channel signal reception systems and equipped with a whole‐body gradient (70‐mT/m maximum strength and 200‐T/m/s maximum slew rate). A custom‐built 16Tx80Rx RF head array with a Food and Drug Administration–approved investigational device exemption was used to acquire human‐brain images. Five healthy adults were scanned who provided a signed informed consent form approved by the local institutional review board before the study session. All calculations including pTx pulse design, image reconstruction, and data analysis were performed in *MATLAB* (MathWorks, Natick, MA, USA) unless noted otherwise.

For pTx pulse design, we obtained calibration data in each volunteer using an in‐house workflow, demonstrated effective at UHF.^32^, ^48^ Briefly, whole‐brain, multichannel B_1_^+^ mapping at 4‐mm isotropic resolution was achieved using a hybrid approach,^49^, ^50^ in which a single absolute volumetric B_1_^+^ map (obtained in the large‐tip‐angle regime by transmitting with all channels) was combined with a series of relative multislice B_1_^+^ maps (obtained in the small‐tip‐angle regime by transmitting one channel at a time). The single absolute B_1_^+^ map was obtained using actual flip‐angle imaging^51^ while operating the RF coil in its circularly polarized (CP) mode. The series of relative B_1_^+^ maps was obtained using multislice two‐dimensional (2D) GRE. Whole‐brain B_0_ mapping at 4‐mm isotropic resolution was achieved based on dual‐echo multislice 2D GRE using the vendor‐provided field‐mapping sequence. Whole‐brain masking was obtained by collecting 2D multislice GRE images at 1‐mm isotropic resolution and applying the FSL's brain extraction tool^52^ to extract the entire brain (including cerebrum, brainstem, and cerebellum). The final calibration data included 16‐channel B_1_^+^ maps, a B_0_ map, and a brain mask, all spanning the entire brain at 4‐mm isotropic resolution over 48 contiguous axial slices. The calibration data taken from seven equidistant slices (20 mm apart from each other) were further down‐sampled to 8‐mm isotropic in‐plane resolution for subsequent pTx pulse design.

We designed subject‐specific pTx spatial‐spectral RF pulses in a way similar to our previous study^46^ to achieve uniform water‐selective excitation across the brain. Briefly, pTx pulses with kT‐point parameterization^53^ were calculated to achieve a desired excitation target in both spatial and spectral domains. The excitation target in the spatial domain dictated nonselective uniform excitation across the brain, whereas that in the spectral domain dictated a water passband (centered at 0 Hz) and a fat stopband (centered at −1565 Hz, the fat‐water frequency shift at 10.5 T), both defined on three points over a bandwidth of 200 Hz in steps of 100 Hz. Channel‐wise RF magnitudes and phases for individual kT points were calculated by solving regularized magnitude least‐squares minimization.^54^ The final pTx pulses were 3.8 ms in length, with 24 kT points constrained to be symmetric about the origin of the 3D excitation k‐space.

For each volunteer, we collected whole‐brain rs‐fMRI data in two runs. During each run, the volunteer was not instructed explicitly to stay still but was asked to fix at crosshairs centered on a gray background projected onto a screen. Each run was acquired using a custom‐built, pTx‐enabled, motion‐robust 3D‐GRE‐EPI sequence with navigators and relevant imaging parameters being scan time = ˜6 min, resolution = isotropic 1.58 mm, field of view (FOV) = 256 × 237 × 190 mm^3^, orientation = sagittal, repetition time (TR)/echo time (TE) = 39/21 ms, flip angle (FA) = 10°, volume TR = 2.34 s, bandwidth = 385.6 kHz, number of interleaves = 2 (i.e., two shots per *k*_*z*_ plane), 2D acceleration rate = 3 × 4 with CAIPI,^55^ and image volumes = 150. The two runs were obtained with opposite phase‐encoding directions: one with anterior–posterior (AP) and another posterior–anterior (PA) phase‐encoding directions to reduce susceptibility‐induced signal dropout in the combined time series,^31^, ^32^ leading to a total of 300 image volumes acquired in about 12 min.

The embedded volumetric navigator images meant for motion and B_0_ tracking were collected between the RF excitation and the beginning of the BOLD signal readout using a multishot 3D EPI method as described in our previous work.^39^ Specifically, for improved time efficiency, accelerated single‐echo navigator images were acquired with matrix size = 48 × 32 × 24, spatial resolution = 5.3 × 7.4 × 7.9 mm^3^, and acceleration rate = 4 × 2 with blipped CAIPI.^56^ Moreover, our navigators were acquired with matrix size of 48 × 32 × 24 corresponding to low resolution of 5.3 × 7.4 × 7.9 mm^3^ when imaging the same FOV as in our high‐resolution fMRI acquisition. The choice of such matrix size was driven by achieving two goals: (i) a TE of about 20 ms for BOLD contrast (by minimizing navigator acquisition time per TR) and (ii) navigators with a temporal resolution better than about 0.5 s (by reducing phase encodes in the slice direction). Within each TR, eight *k*_*x*_ lines were acquired in the *k*_*y*_‐*k*_*z*_ plane following the prescribed 2D CAIPI pattern. With an echo spacing of 0.47 ms, the navigator acquisition was less than 5 ms within each TR. An accelerated navigator image volume was acquired in 12 successive TRs, leading to a temporal resolution of 468 ms. As in our previous work,^39^ two blipless readout lines at the center of k‐space used to correct EPI ghost and global B_0_ variation were acquired instead of the navigator once every second.

For each volunteer, we acquired fully sampled calibration data in a separate reference scan using a custom pTx‐enabled, multi‐echo 3D‐GRE sequence. This data were used for estimating multicoil sensitivity maps needed for parallel‐image reconstruction as well as deriving a field map for EPI susceptibility distortion correction in fMRI preprocessing. Specifically, three‐echo GRE images were acquired using the same pTx pulses as used for the BOLD‐fMRI data acquisition. Other relevant imaging parameters were resolution = 4 mm isotropic, TR/TE1/TE2/TE3 = 30/3/3.73/4.46 ms, FA = 12°, matrix size = 74 × 60 × 48, orientation = sagittal, and scan time = ˜1.5 min.

To facilitate subsequent anatomy‐based fMRI processing and analysis, we collected whole‐brain anatomical reference images using a T_1_‐weighted (T_1_w) 3D magnetization–prepared two rapid acquisition gradient echoes (MP2RAGE) sequence.^57^ For each volunteer, the MP2RAGE was acquired at 1‐mm isotropic resolution with CP mode excitation. Other relevant imaging parameters were TR/TE = 5000/1.64 ms, inversion time TI1/TI2 = 1100/2500 ms, FA1/FA2 = 5°/5°, and scan time = ˜7 min.

### 2.2 Image reconstruction

We reconstructed fMRI time series on a volume‐by‐volume basis using in‐house *MATLAB* software. In the image reconstruction, the forward model considered the effect of rigid‐body head motion, spatially linear B_0_ changes, and receive sensitivity maps in the under‐sampled multichannel k‐space data. Multicoil sensitivity maps were estimated from the first echo of the 3D multi‐echo GRE reference scans using an in‐house algorithm.^39^ For both intrascan, rigid‐body head motion and B_0_ measurements, accelerated navigator images were reconstructed using a 2D‐GRAPPA algorithm.^58^ From the navigator magnitude images, motion time series were estimated using an iterative multiresolution image‐registration approach.^59^ B_0_ changes over time were estimated based on the navigator phase images. To study how joint motion and B_0_ correction would affect downstream fMRI analysis, uncorrected reconstruction was also performed for comparison where EPI time series were reconstructed using the same raw data but without motion or B_0_ correction. Details about the implementation of the correction and reconstruction algorithm can be found in our previous publications.^39^, ^40^, ^45^

### 2.3 Data processing

After image reconstruction, we performed BOLD‐fMRI processing and analysis at subject and group levels. This was done by integrating open‐source Brain Imaging Data Structure (BIDS)^60^–based pipelines into a custom‐built workflow to perform structural‐MRI‐facilitated fMRI preprocessing and postprocessing. Briefly, our workflow was devised to fulfill four steps: (i) create BIDS input data structure using dcm2bids^61^ (https://unfmontreal.github.io/Dcm2Bids); (ii) perform structural MRI preprocessing using the *sMRIPrep* pipeline^62^ (https://www.nipreps.org/smriprep); (iii) conduct fMRI preprocessing using the *fMRIPrep* pipeline^63^, ^64^ (https://fmriprep.org/en/latest); and (iv) carry out functional connectivity–related postprocessing (e.g., temporal filtering and confound regression) using the extensible connectivity pipeline‐DCAN (*XCP‐D*)^65^ (https://xcp‐d.readthedocs.io/en/latest/index.html). Details on how each pipeline was run through our workflow are provided subsequently.

Our workflow used MP2RAGE to define the volunteer's native T_1_w space. For compatibility with the *sMRIPrep* pipeline optimized for MPRAGE, MP2RAGE‐combined images were made more like MPRAGE (by removing background noise) using tools shared at https:// github.com/srikash/3dMPRAGEise. The structural MRI preprocessing with the *sMRIprep* pipeline was performed as follows. First, the T_1_w images were corrected for intensity nonuniformity with N4BiasFieldCorrection,^66^ distributed with *ANTs 2.5.4* ^67^ (RRID:SCR_004757) and used as T_1_w reference throughout the workflow. The T_1_w reference was then skull‐stripped with a *Nipype* implementation of the antsBrainExtraction.sh workflow (from *ANTs*) using *OASIS30ANTs* as target template. Brain‐tissue segmentation of cerebrospinal fluid (CSF), white matter (WM), and gray matter (GM) was performed on the brain‐extracted T_1_w using fast from *FSL*.^68^ Volume‐based spatial normalization to one standard space (MNI152NLin2009cAsym) was performed through nonlinear registration with antsRegistration (*ANTs* 2.5.4) using brain‐extracted versions of both T_1_w reference and the T_1_w template. The following template was selected for spatial normalization and accessed with *TemplateFlow* ^69^ (24.2.0): *ICBM 152 Nonlinear Asymmetrical template version 2009c* ^70^ (RRID:SCR_008796; *TemplateFlow* ID: MNI152NLin2009cAsym).

Using outputs from the *sMRIPrep* pipeline, the fMRI preprocessing with the *fMRIPrep* pipeline was carried out as follows for functional data alignment and normalization. Brain surfaces were reconstructed using recon‐all (*FreeSurfer* 7.3.2,^71^ RRID:SCR_001847), and the brain mask estimated previously was refined with a custom variation of the method to reconcile *ANTs*‐derived and *FreeSurfer*‐derived segmentations of the cortical GM of *Mindboggle* ^72^ (RRID:SCR_002438). Grayordinate “dscalar” files containing 91 k samples were resampled onto *fsLR* using the Connectome Workbench.^73^

Susceptibility‐induced distortions in the BOLD images were corrected using field map–based correction workflows implemented via ***sdcflows***,^74^ a component of the *fMRIPrep* pipeline. Head‐motion parameters with respect to the BOLD reference are estimated before any spatiotemporal filtering using mcflirt from *FSL*.^75^ The BOLD reference was then coregistered to the T_1_w reference using bbregister from *FreeSurfer*, which implements boundary‐based registration.^76^ Coregistration was configured with six degrees of freedom. Several confounding time series were calculated based on the preprocessed BOLD: framewise displacement (FD), derivative of root mean square variance over voxels (DVARS), and three region‐wise global signals from CSF, WM, and whole brain. FD and DVARS are calculated for each functional run both using their implementations in *Nipype*.^77^ The three global signals are extracted within the CSF, the WM, and the whole‐brain masks. Additionally, a set of physiological regressors were extracted to allow for component‐based noise correction^78^ (CompCor). Principal components are estimated after high‐pass‐filtering the preprocessed BOLD time series (using a discrete cosine filter with 128‐s cut‐off) for the two *CompCor* variants: temporal (tCompCor) and anatomical (aCompCor). The tCompCor components are then calculated from the top 2% variable voxels within the brain mask. For aCompCor, three probabilistic masks (CSF, WM, and combined CSF + WM) are generated in anatomical space. The implementation differs from that of Behzadi et al. in that instead of eroding the masks by 2 pixels on BOLD space, a mask of pixels that likely contains a volume fraction of GM is subtracted from the aCompCor masks. This mask is obtained by dilating a GM mask extracted from *FreeSurfer*'s *aseg* segmentation, and it ensures components are not extracted from voxels containing a minimal fraction of GM. Finally, these masks are resampled into BOLD space and binarized by thresholding at 0.99 (as in the original implementation). Components are also calculated separately within the WM and CSF masks. For each CompCor decomposition, the *k* components with the largest singular values are retained, such that the retained components' time series are sufficient to explain 50% of variance across nuisance masks (CSF, WM, combined, or temporal). The remaining components are dropped from consideration. The head‐motion estimates calculated in the correction step were also expanded with the inclusion of temporal derivatives and quadratic terms for each.^79^ Frames that exceeded a threshold of 0.5‐mm FD or 1.5 standardized DVARS were annotated as motion outliers. Additional nuisance time series are calculated as the framewise displacement across head‐motion parameters. All 91 k fsaverage “grayordinate” time series were resampled onto the left/right‐symmetric space “fsLR” using the Connectome Workbench.^73^ Grayordinates files^73^ containing 91 k samples were also generated with surface data transformed to 2‐mm resolution using the MNI152NLin6Asym space. All resamplings can be performed with a single interpolation step by composing all the pertinent transforms (i.e., head‐motion transform, anatomical coregistration when available, and coregistrations to standard and output spaces). Gridded (volumetric) resamplings were performed using nitransforms, configured with the B‐spline interpolation.

Using the preprocessed BOLD data, temporal SNR (tSNR) maps were calculated at subject and group levels. For each volunteer, the two‐per‐run tSNR maps (both defined as the mean of time series divided by its standard deviation at each voxel, i.e., mean[voxel time series] / std[voxel time series]^80^) were first calculated by considering all the preprocessed 150 image volumes in each run, and were then averaged to form the subject's tSNR map in the standard MNI volume space. The group tSNR map was created by averaging subjects' tSNR maps across all 5 volunteers. In each case, the whole‐brain tSNR histograms of both motion‐corrected and uncorrected reconstructions were created for comparison.

The fMRI postprocessing with the *XCP‐D* pipeline was conducted as follows. In total, 36 nuisance regressors were selected from the preprocessing confounds, according to the “36P” strategy, including six motion parameters, mean global signal, mean WM signal, mean CSF signal with their temporal derivatives, and quadratic expansion of six motion parameters, tissue signals, and their temporal derivatives.^79^, ^81^ The BOLD data were converted to NIfTI format, despiked with *AFNI's* 3dDespike, and converted back to CIFTI format. Nuisance regressors were regressed from the BOLD data using a denoising method based on Nilearn's approach. The time series were band‐pass‐filtered using a second‐order Butterworth filter to retain signals between 0.01 and 0.08 Hz. The same filter was applied to the confounds. The resulting time series were then denoised using linear regression.

The amplitude of low‐frequency fluctuation (ALFF)^82^ was computed by transforming the mean‐centered, standard deviation–normalized, denoised BOLD time series to the frequency domain. The power spectrum was computed within the 0.01–0.08‐Hz frequency band, and the mean square root of the power spectrum was calculated at each voxel to yield voxel‐wise ALFF measures. The resulting ALFF values were then multiplied by the standard deviation of the denoised BOLD time series to retain the original scaling. For each hemisphere, regional homogeneity (ReHo)^83^ was computed using surface‐based 2dReHo.^84^ Specifically, for each vertex on the surface, the Kendall's coefficient of concordance (KCC) was computed with nearest‐neighbor vertices to yield ReHo. For the subcortical, volumetric data, ReHo was computed with neighborhood voxels using 3dReHo from *AFNI*.^85^

For each volunteer and given reconstruction, the two‐per‐run ALFF/ReHo maps were first calculated based on all the postprocessed 150 image volumes in each run and then averaged to produce the final ALFF/ReHo map across the standard grayordinate space (including cortical surfaces and subcortical voxels). Group ALFF/ReHo maps across the standard grayordinate space^73^ were also obtained by averaging across all 5 volunteers (a total of 10 resting‐state fMRI runs). All ALFF/ReHo maps were visualized using the Connectome Workbench^86^ (https://www.humanconnectome.org/software/workbench‐command). In each case, the whole‐grayordinate‐space ALFF/ReHo histograms of both motion‐corrected and uncorrected reconstructions were created for comparison.

## 3 RESULTS

**FIGURE 1:**
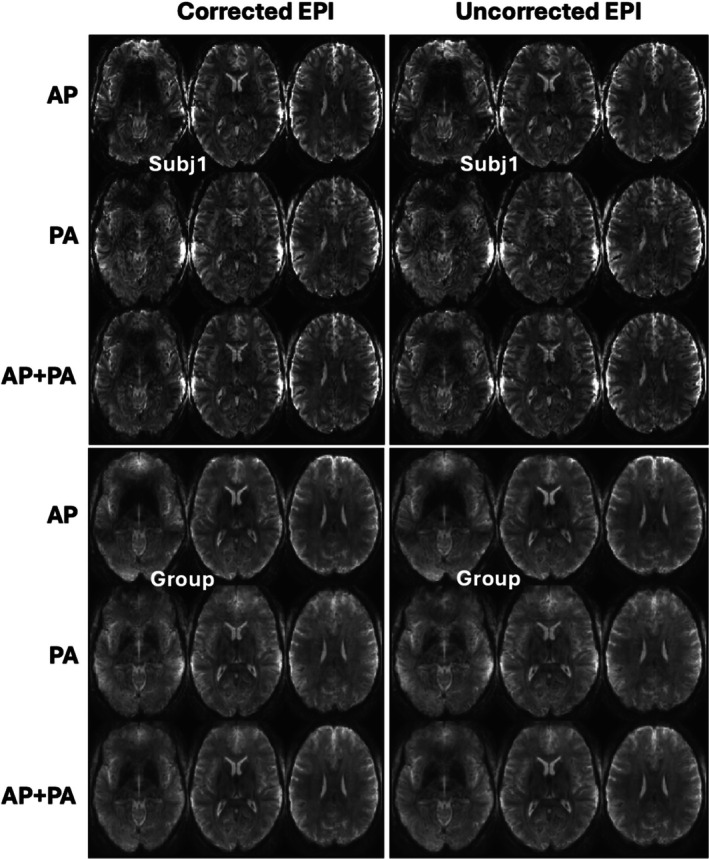
Accelerated four‐dimensional (4D) mean images (i.e., averaged in the time dimension) of blood oxygen level–dependent (BOLD) echo‐planar imaging (EPI) time series for reconstruction with motion and field correction (Corrected EPI) versus reconstruction using the same raw data but with no correction (Uncorrected EPI) at both subject and group levels. Shown are 4D mean images for anterior–posterior (AP) phase‐encoding acquisition only, for posterior–anterior (PA) phase‐encoding acquisition only, and for combining both phase‐encoding acquisitions (AP + PA). Images from 1 representative volunteer and at the group level (average across 5 volunteers) are shown here for demonstration. For each volunteer, two resting‐state functional MRI runs were acquired: one with AP and another with PA phase encodes, both collected at 1.58‐mm isotropic resolution using a motion‐robust three‐dimensional EPI sequence with 21‐ms echo time, 2.34‐s volume repetition time, 12‐fold combined acceleration and 150 volumes, leading to a total of 300 volumes acquired in about 12 min. Furthermore, multi‐echo gradient‐echo images at 4‐mm isotropic resolution were collected separately to estimate coil sensitivities for image reconstruction and to derive a field map to inform EPI susceptibility distortion correction in the preprocessing. In all cases, subject‐specific parallel‐transmit radiofrequency (RF) pulses were designed for uniform water‐selective excitation across the brain, and data were acquired using a custom‐built, 16‐channel transmit, 80‐channel receive head RF coil. The reconstructed BOLD time series went through custom‐built, fMRIPrep‐based preprocessing pipelines, including susceptibility distortion correction, coregistration, and confound identification. Note that for both corrected and uncorrected reconstructions, the combination of the AP and PA images resulted in minimal signal dropout, thereby reducing the effect of off resonances at both subject and group levels.

For both motion‐corrected and uncorrected reconstructions, the preprocessed BOLD time series were of high quality (Figure 1) at both subject and group levels. Although signal dropout due to susceptibility was observed in both acquisitions with AP and PA phase‐encoding directions, the combination of the two led to little signal dropout. Furthermore, for each volunteer (Figure S1), the EPI susceptibility distortions were effectively corrected by using the fMRIPrep preprocessing pipelines. The unprocessed images from 1 volunteer are also shown in Figure S2 to provide a sense of how the raw images looked like.

**FIGURE 2:**
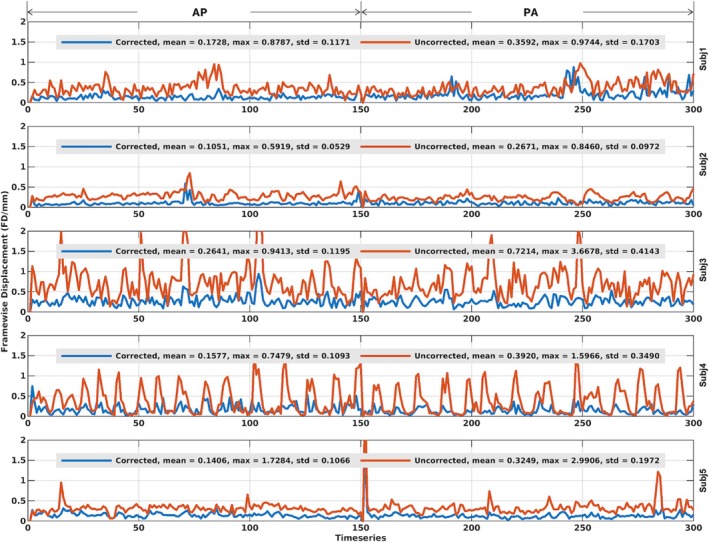
Framewise displacement (FD) for reconstruction with motion and field correction (Corrected) versus reconstruction using the same raw data but with no correction (Uncorrected) at the subject level. Shown are time courses of standardized FD values in millimeters (output from the fMRIPrep pipeline) for Corrected (*blue*) versus Uncorrected (*red*) reconstruction. For each volunteer and given reconstruction, the FD time course shown was that obtained from the anterior–posterior phase‐encoding acquisition (the first half of the entire time course), followed by that from the posterior–anterior phase‐encoding acquisition (the second half of the entire time course). Note how the reconstruction with motion and field correction effectively reduced FD values in every volunteer. std, standard deviation.

Our motion‐corrected reconstruction largely improved image quality for BOLD time series (Figure 2), reducing motion confounds as assessed by the FD measurement. Reduction in FD values were observed in every volunteer. Quantitatively, at the subject level, mean FD values were reduced by a factor ranging from about 53% (0.17 vs. 0.36 for uncorrected reconstruction in Volunteer 1) up to about 64% (0.26 vs. 0.72 for uncorrected reconstruction in Volunteer 3), and maximum FD values reduced by a factor ranging from about 9% (0.88 vs. 0.97 for uncorrected reconstruction in Volunteer 1) up to about 74% (0.94 vs. 3.67 for uncorrected reconstruction in Volunteer 3).

**FIGURE 3:**
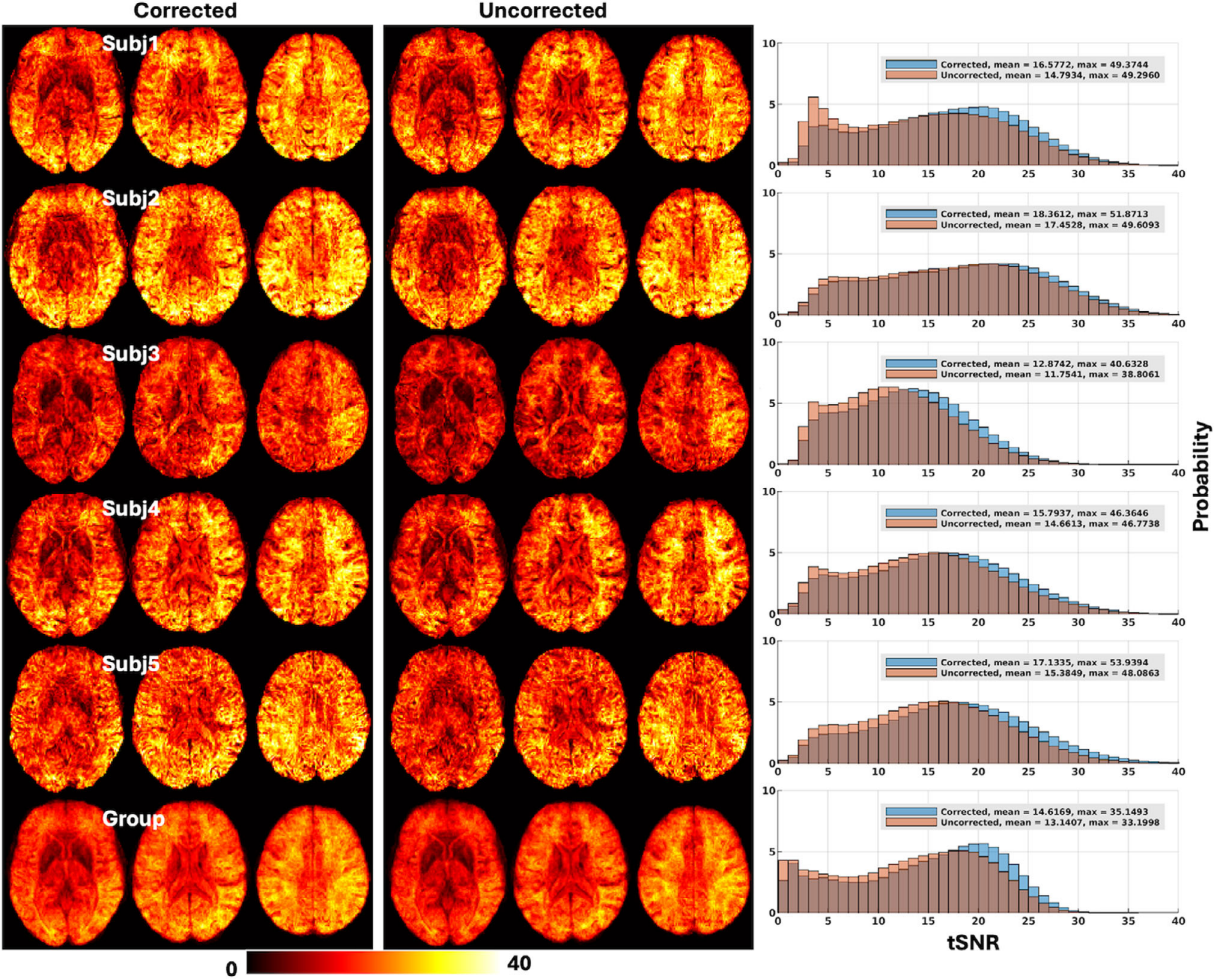
Temporal SNR (tSNR) maps calculated using preprocessed blood oxygen level–dependent data (with volume‐wise motion correction applied) for reconstruction with motion and field correction (Corrected) versus reconstruction using the same raw data but with no correction (Uncorrected). Shown are tSNR maps in three representative axial slices for Corrected (*leftmost panel*) versus Uncorrected (*middle panel*) reconstruction at subject and group levels, along with associated whole‐brain tSNR histograms (*rightmost panel*). For each volunteer and given reconstruction, the two per‐run tSNR maps were first calculated by considering all the preprocessed 150 image volumes in each run and were averaged to form the final tSNR map in the standard MNI volume space. The group tSNR map was created by averaging tSNR maps of each individual across 5 volunteers. In each case, the tSNR histograms of the two reconstructions were created for comparison (where the vertical axis is “Probability” defined as the number of observations in bin divided by the total number of observations), along with associated mean and max tSNR values reported. Note the tSNR improvement with motion and field correction at both subject and group levels.

In addition to the reduction in FD values, an improvement in tSNR was observed at both subject and group levels (Figure 3). At the subject level, mean tSNR values averaged across the brain increased by a factor ranging from bout 5% (18.4 vs. 17.5 for uncorrected reconstruction in Volunteer 2) up to about 12% (16.6 vs. 14.8 for uncorrected reconstruction in Volunteer 1). At the group level, the mean tSNR value averaged across all 5 volunteers increased by about 11% (14.6 vs. 13.1 for uncorrected reconstruction).

**FIGURE 4:**
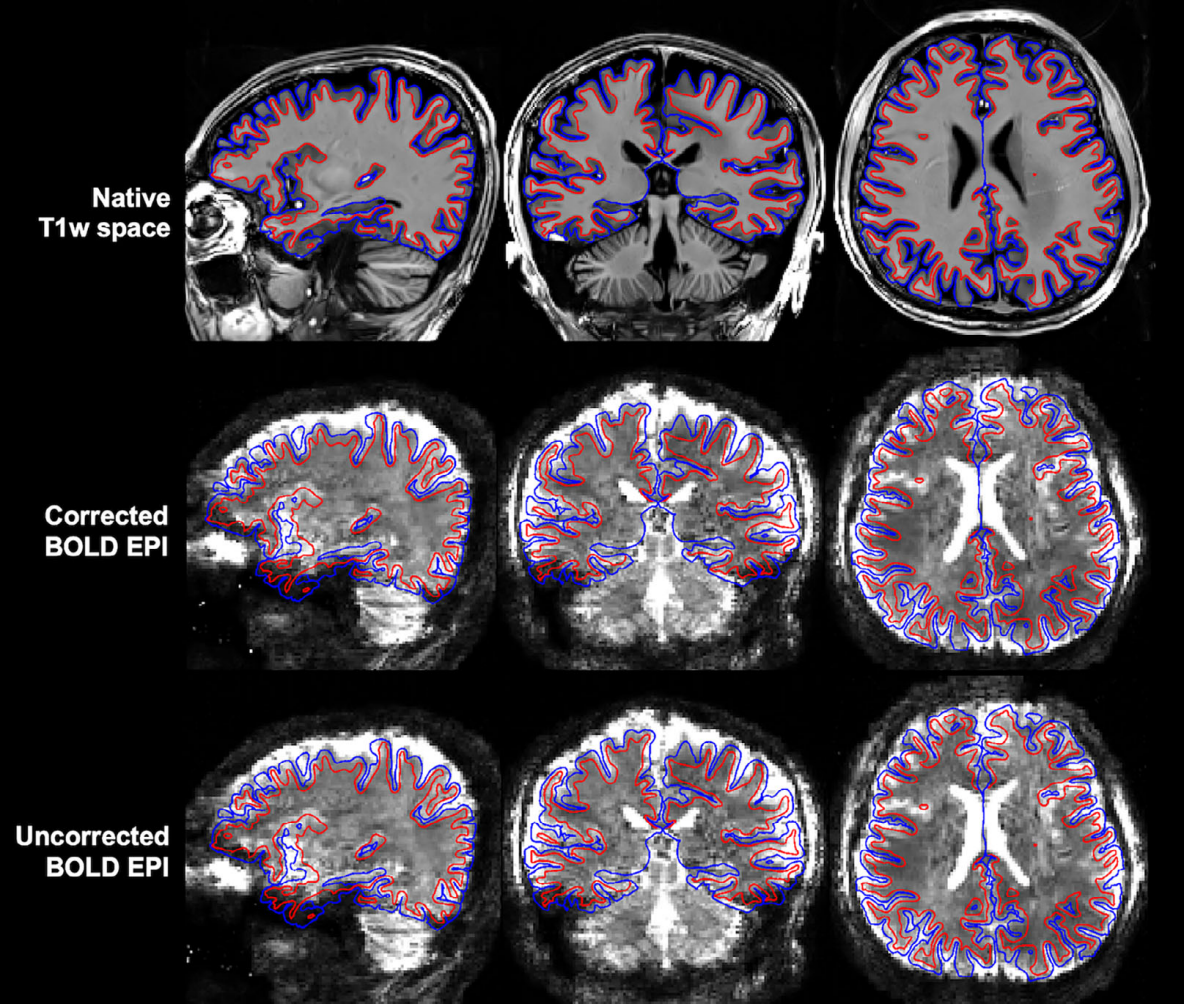
Preprocessing of data acquisition at 10.5 T. Shown are results for a representative volunteer including white matter (*red*) and pial (*blue*) surfaces overlaid on the volunteer's native T_1_‐weighted (T1w) images (*top*), four‐dimensional (4D) mean of coregistered blood oxygen level–dependent (BOLD) echo‐planar imaging (EPI) time series reconstructed with motion and field correction (*middle*), and 4D mean of coregistered BOLD‐EPI time series reconstructed using the same EPI raw data but without motion or field correction (*bottom*). The native T1w space was defined by collecting whole‐brain magnetization‐prepared two rapid gradient‐echo (MR2RAGE) images at 1‐mm isotropic resolution. The 4D mean images shown were obtained combining both anterior–posterior and posterior–anterior phase‐encoding acquisitions. Note that good surface reconstruction and coregistration were achieved despite the B_1_^+^and B_0_ challenges at 10.5 T.

Despite B_1_^+^and B_0_ challenges encountered at 10.5 T, satisfactory surface reconstruction and coregistration (Figure 4) were achieved. Using MP2RAGE T_1_w structural images to delineate cortical ribbons for surface analysis resulted in good definition of WM and pial surfaces nearly across the entire brain. For both motion‐corrected and uncorrected reconstructions, the BOLD time series were effectively coregistered to the volunteers' native T_1_w space defined by the MP2RAGE images, with the WM surface following the GM and WM boundary and the pial surface tracing the outer edge of the GM in most brain regions.

**FIGURE 5:**
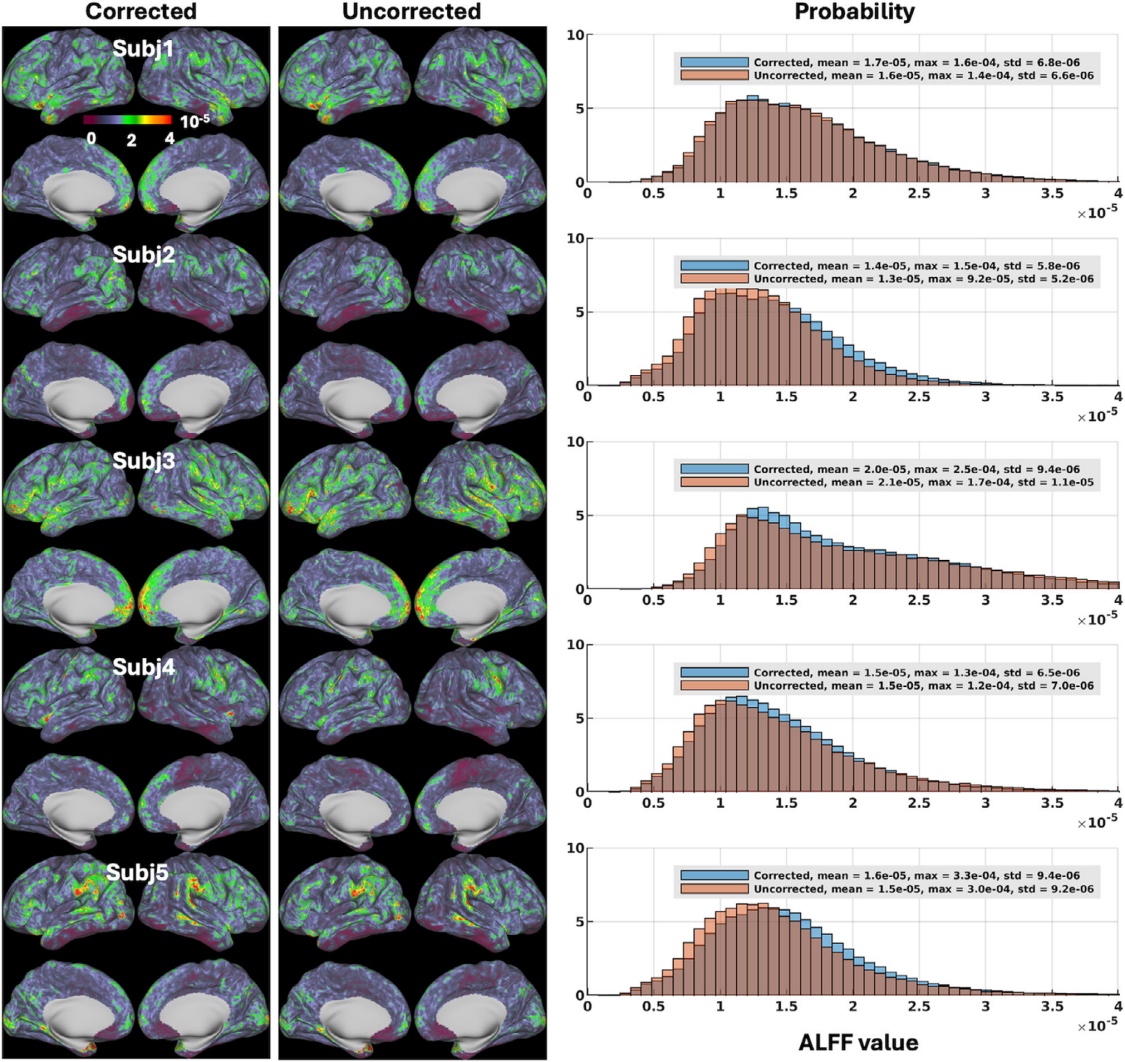
Amplitude of low‐frequency fluctuation (ALFF) maps for reconstruction with motion and field correction (Corrected) versus reconstruction using the same raw data but with no correction (Uncorrected) at the subject level. Shown are ALFF maps on inflated cortical surfaces for Corrected (*leftmost panel*) versus Uncorrected (*middle panel*) reconstruction, along with associated whole‐grayordinate‐space ALFF histograms (*rightmost panel*). For each volunteer and given reconstruction, the two per‐run ALFF maps were first calculated based on all the postprocessed 150 image volumes in each run and were averaged to produce the final ALFF map across the standard grayordinate space (including cortical surfaces and subcortical voxels). Data postprocessing was performed at the run level using the XCP‐D pipeline for despiking, band‐pass filtering, and denoising. ALFF was calculated from the power spectrum of the postprocessed functional time series within the 0.01–0.08‐Hz frequency band. In each volunteer, the ALFF histograms of the two reconstructions were created for comparison (where the vertical axis is “Probability” defined as the number of observations in bin divided by the total number of observations) along with associated mean and maximum ALFF values reported. Note how image reconstruction with motion and field correction affected ALFF evaluation, overall increasing ALFF values in most volunteers. std, standard deviation.

**FIGURE 6:**
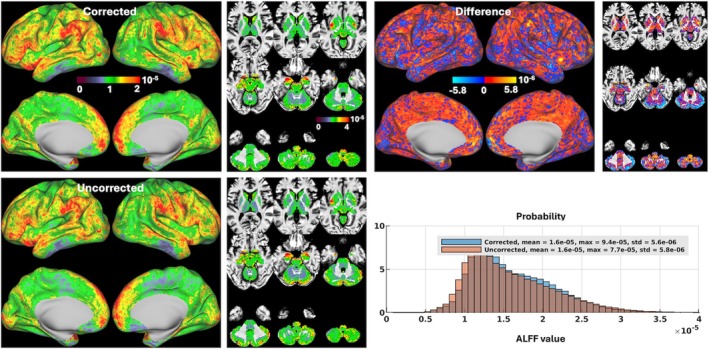
Amplitude of low‐frequency fluctuation (ALFF) maps for reconstruction with motion and field correction (Corrected) versus reconstruction using the same raw data but with no correction (Uncorrected) at the group level. Shown are group ALFF maps across the standard grayordinate space for Corrected versus Uncorrected reconstructions, averaged across the 5 volunteers (a total of 10 resting‐state functional MRI runs), visualized on inflated cortical surfaces (*left panel*) and in subcortical voxels overlaid on T_1_‐weighted structural images in the MNI standard volume space (*right panel*); also shown is the difference map of the two reconstructions, calculated as Corrected ALFF minus Uncorrected ALFF. In addition, the reconstruction‐specific histograms of group ALFF across the entire grayordinate space were also created for comparison (where the vertical axis is “Probability” defined as the number of observations in bin divided by the total number of observations). Note how image reconstruction with motion and field correction overall increased ALFF at the group level. std, standard deviation.

Our motion‐corrected reconstruction also led to changes in ALFF mapping at both subject and group levels. At the subject level (Figure 5), both mean ALFF values averaged and maximum ALFF values obtained across the entire standard grayordinate space were mostly increased, with the mean ALFF values being increased in 3 volunteers by up to about 8% (1.4e‐5 vs. 1.3e‐5 for uncorrected reconstruction in Volunteer 2) and the maximum ALFF values being increased in all 5 volunteers by up to about 63% (15e‐5 vs. 9.2e‐5 for uncorrected reconstruction in Volunteer 2). At the group level (Figure 6), ALFF values were increased in most cortical regions and subcortical nuclei, with the maximum ALFF value being increased by about 22% (9.4e‐5 vs. 7.7e‐5 for uncorrected reconstruction).

**FIGURE 7:**
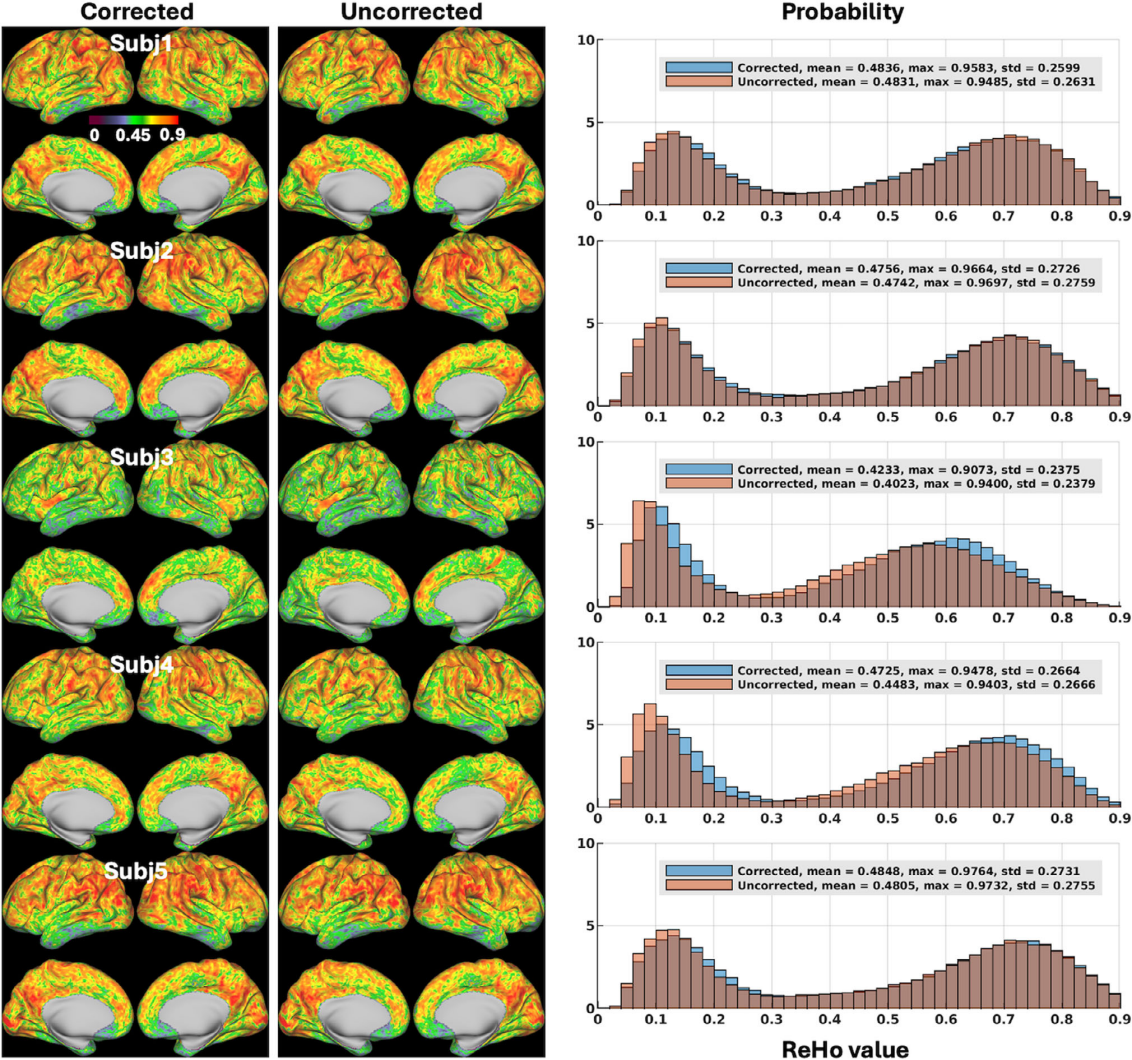
Regional homogeneity (ReHo) maps for reconstruction with motion and field correction (Corrected) versus reconstruction using the same raw data but with no correction (Uncorrected) at the subject level. Shown are ReHo maps on inflated cortical surfaces for Corrected (*leftmost panel*) versus Uncorrected (*middle panel*) reconstruction, along with associated whole‐grayordinate‐space ReHo histograms (*rightmost panel*). For each volunteer and given reconstruction, the two per‐run ReHo maps were first calculated based on all the postprocessed 150 image volumes in each run and were averaged to produce the final ReHo map across the standard grayordinate space (including cortical surfaces and subcortical voxels). Data postprocessing was performed at the run level using the XCP‐D pipeline for despiking, band‐pass filtering, and denoising. Cortical ReHo values were computed using a surface‐based two‐dimensional algorithm (2dReHo), whereas subcortical ReHo values were computed using AFNI's 3dReHo. In each volunteer, the ReHo histograms of the two reconstructions were created for comparison (where the vertical axis is “Probability” defined as the number of observations in bin divided by the total number of observations) along with associated mean and maximum ReHo values reported. Note how image reconstruction with motion and field correction affected ReHo evaluation, overall increasing ReHo values especially in high‐motion volunteers. std, standard deviation.

**FIGURE 8:**
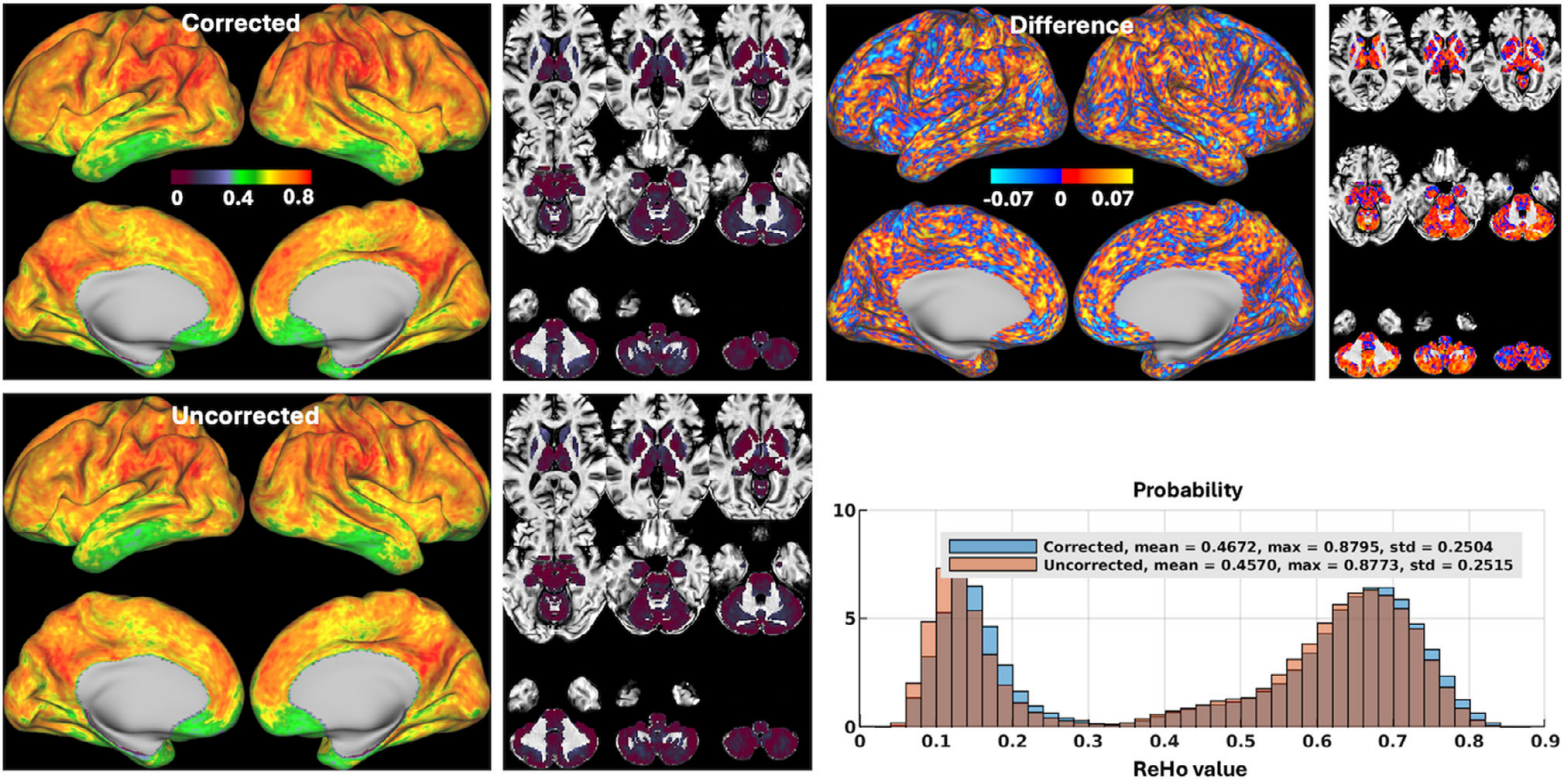
Regional homogeneity (ReHo) maps for reconstruction with motion and field correction (Corrected) versus reconstruction using the same raw data but with no correction (Uncorrected) at the group level. Shown are group ReHo maps across the standard grayordinate space for Corrected versus Uncorrected reconstructions, averaged across the 5 volunteers (a total of 10 resting state fMRI runs), visualized on inflated cortical surfaces (*left panel*) and in subcortical voxels overlaid on T_1_‐weighted structural images in the MNI standard volume space (*right panel*); also shown is the difference map of the two reconstructions, calculated as Corrected ReHo minus Uncorrected ReHo. In addition, the reconstruction‐specific histograms of group ReHo across the entire grayordinate space were also created for comparison (where the vertical axis is “Probability” defined as the number of observations in bin divided by the total number of observations). Note how image reconstruction with motion and field correction overall increased ReHo at the group level. std, standard deviation.

Likewise, our motion‐corrected reconstruction gave rise to changes in ReHo mapping at both subject and group levels. At the subject level (Figure 7), both mean ReHo values averaged and maximum ReHo values obtained across the entire standard grayordinate space were mostly increased, with the mean ReHo values being increased in all 5 volunteers by up to about 6% (0.473 vs. 0.448 for uncorrected reconstruction in Volunteer 4) and the maximum ReHo values being increased in 3 volunteers by up to about 1% (0.958 vs. 0.949 for uncorrected reconstruction in Volunteer 1). At the group level (Figure 8), ReHo values were increased in most subcortical voxels (including subcortical nuclei and cerebellum), with the mean ReHo value being increased by about 2% (0.467 vs. 0.457 for uncorrected reconstruction).

## 4 DISCUSSION

We have successfully demonstrated the feasibility of quality whole‐brain BOLD fMRI in humans at 10.5 T. Pivotal to this success was the development of a new comprehensive imaging method by integrating various cutting‐edge techniques, including motion‐robust 3D GRE EPI, parallel transmission, and high‐density RF receive coils. We demonstrated the utility of our new method by acquiring resting‐state fMRI data (at 1.58‐mm isotropic resolution with approximately 2.3‐s volume TR) in healthy volunteers using a custom‐built 16‐channel transmit, 80‐channel receive RF head array and by performing image analysis relevant to functional connectivity estimation. Our results showed that our new method gave rise to quality BOLD time series (Figure 1), reducing motion confounds (Figure 2). The reduction in motion confounds translated into enhanced tSNR (Figure 3) and increased ALFF and ReHo values (Figures 5, 6, 7, 8) at both subject and group levels.

Instead of using a traditional approach with conventional binomial water‐selective excitation pulses applied in the CP mode, we designed pTx spatial‐spectral pulses for achieving uniform water‐selective excitation across the entire brain. Indeed, the use of our pTx pulses was found to outperform the traditional approach (Figure S3), largely improving FA uniformity while effectively eliminating RF shading artifacts. Quantitative analysis based on Bloch simulation further reveals that the use of our pTx pulses can reduce the coefficient of variation (i.e., standard deviation/mean) of FA distribution across the entire brain by up to about 48% (17% vs. 33% for the CP mode). One drawback of the pTx pulse design used here is that the water‐selective excitation pulses were designed by solving a regularized magnitude least‐squares problem with total RF power control. Part of our future work is to design such pulses with explicit local specific‐absorption‐rate constraints using a more comprehensive framework^87^ to maximize pTx performances for a given imaging protocol.

Results in Figure 2 show that our motion‐corrected image reconstruction can substantially reduce the amplitudes of FD time courses especially in high‐motion volunteers, illustrating that it can help reduce the volume misalignment (due to intervolume head motion) to be corrected by the spatial coregistration performed in fMRI preprocessing. Our motion‐corrected image reconstruction was found to also alter time courses (Figure S4) of DVARS^88^ (an index commonly used to dictate the rate of temporal change in BOLD signal across the entire brain at each frame of data) when calculated on the preprocessed BOLD time series. For each volunteer, we also quantified the correlation of the DVARS and FD time courses in two scenarios using the DVARS time course (i) from the motion‐corrected reconstruction and (ii) from the uncorrected reconstruction. In both scenarios, the FD time course from the uncorrected reconstruction was used, as it closely reflected the intervolume rigid‐body head motion incurred during the fMRI scan. Our quantification (Table ure S1) indicated that our motion‐corrected reconstruction reduced the correlation of the DVARS and FD time courses in every volunteer, with the correlation averaged across volunteers reduced by about 35% (0.32 vs. 0.49 for uncorrected reconstruction). This indicates that our motion‐corrected reconstruction can decrease the effect of head motion on BOLD signal.^89^

For evaluation of our motion‐robust 3D‐EPI method, we calculated ALFF^90^ and ReHo,^91^ two rs‐fMRI metrics commonly used to measure spontaneous neural activity and local temporal similarity in the BOLD data, respectively. Our results at the group level (Figures 6 and 8) showed that our motion‐corrected image reconstruction led to higher ALFF and ReHo values in brain areas within the default mode network (including the medial prefrontal cortex and bilateral inferior parietal lobule), consistent with previous studies.^82^, ^90^, ^92^ When compared with the uncorrected reconstruction, our motion‐corrected reconstruction increased ALFF values in most cortical surfaces and deep brain nuclei (Figure 6), suggesting that correcting for both rigid‐body head motion and B_0_ field changes through image reconstruction can help enhance the amplitude of low‐frequency fluctuation in BOLD signal, thereby improving the detection of spontaneous neural activity. Moreover, our motion‐corrected reconstruction was found to reduce the artificially high ALFF values along the outer boundary of cerebellum GM observed when using the uncorrected reconstruction. Those artificially high ALFF values are believed to result from contamination of surrounding CSF (via partial volume effects), which is reported^82^, ^90^ to have artificially high ALFF values due to physiological noise (e.g., arising from respiratory‐volume variability^93^). Likewise, our motion‐corrected reconstruction enhanced ReHo (Figure 8) especially in subcortical regions (including cerebellum) where the ReHo values were found much lower than over cortical surfaces, indicating that correcting for motion and B_0_ field changes early in image reconstruction can improve the synchrony of BOLD signal in neighboring voxels, thereby promoting the characterization of network centrality of local functional connectivity.^83^

To examine how our motion‐corrected image reconstruction would alter the estimation of functional connectivity, we divided the 5 volunteers into two groups based on their head motion: (i) the low‐motion group (consisting of Volunteers 1, 2, and 5) and (ii) the high‐motion group (including Volunteers 3 and 4). For each group, we created the functional connectivity matrix by computing Pearson's correlations and compared it with that of uncorrected reconstruction. Our result (Figure S5) showed that although our motion‐corrected reconstruction had an effect in both low‐motion and high‐motion groups, the effect became larger for the high‐motion group, leading to greater alterations in the estimation of functional connectivity. Quantitatively, the average alteration (calculated as the mean of absolute values of differences in functional connectivity between our motion‐corrected and uncorrected reconstructions) increased by about 56%, going from 0.073 for the low‐motion group to 0.114 for the high‐motion group. We also found that our motion‐corrected reconstruction decreased the differences in functional connectivity between the low‐motion and high‐motion groups, reducing the mean of absolute differences by about 8% (0.152 vs. 0.165 for uncorrected reconstruction).

The tSNR comparison reported in Figure 3 was based on calculation using preprocessed BOLD data (e.g., with volume realignment, susceptibility distortion correction, and normalization being applied via fMRIPrep pipelines). To isolate the direct effects of our motion‐corrected reconstruction from those of preprocessing, we calculated tSNR using unprocessed BOLD data (i.e., before fMRI preprocessing). Our results (Figure S6) showed that our motion‐corrected reconstruction increased whole‐brain average tSNR at the subject level by a factor ranging from bout 14% (15.9 vs. 14.0 for uncorrected reconstruction in Volunteer 2) up to about 38% (10.2 vs. 7.4 for uncorrected reconstruction in Volunteer 3) and at the group level by about 24% (10.7 vs. 8.6 for uncorrected reconstruction).

Here our motion‐corrected reconstruction incorporated simultaneous correction of rigid‐body head motion and B_0_ field changes. To quantify the individual contributions of motion correction and field correction to the overall tSNR gain, we performed additional reconstruction using the same raw data but now with field‐only correction (i.e., without rigid‐body head motion correction per se). The associated tSNR was assessed using preprocessed BOLD data and compared with those for uncorrected and motion‐corrected reconstructions. Our results (Figure S7) showed that reconstruction with field‐only correction increased whole‐brain average tSNR by about 6% at the group level when compared with uncorrected reconstruction. Given the approximate 11% overall tSNR gain achieved at the group level (Figure 3), this indicates that the rigid‐body head motion correction also performed in our motion‐corrected reconstruction on average contributed an additional 5% tSNR gain. This approximate 5% tSNR gain is believed to be attributed to the correction of intravolume rigid‐body head motion, which cannot be addressed with volume realignment in fMRI preprocessing.

To further evaluate the performance of our motion‐corrected reconstruction for intervolume motion correction, we analyzed the motion parameters estimated from retrospective volume realignment in fMRI preprocessing. Particularly, we quantified the root mean square (RMS) values for the six rigid‐body motion parameters, which should be viewed as a measure of residual volume misalignment left over from our motion‐corrected reconstruction. The RMS values thus can also be regarded as a measure of error (i.e., RMSE) in our estimation of intervolume motion parameters based on volume realignment of low‐resolution navigator images in reference to accurate intervolume motion estimation based on volume realignment of high‐resolution fMRI images from uncorrected reconstruction. Our results (Figure S8) showed that our motion‐corrected reconstruction substantially reduced the amount of volume misalignment to be corrected in fMRI preprocessing, with estimation error on average being under about 0.2 mm for translational motion and under about 0.15 degrees for rotational motion.

To examine potential effects of high‐resolution fMRI sampling on our navigator‐based motion and field estimation and to avoid any physiological confounds, we conducted a phantom study in which a head‐shaped water phantom was scanned using the same RF coil as in our human scans. Specifically, we collected two navigator data sets with identical navigator parameters (e.g., FOV, resolution, k‐space sampling): (i) one using the same imaging protocol as in our human scan and (ii) another using the same sequence as in Data Set 1 but followed by minimal EPI readout gradients. Both navigator data sets were then used to estimate motion and field parameters as in our motion‐corrected reconstruction. Our results (Figure S9) showed that both navigator data sets led to similar estimates of motion and field parameters, with subtle differences observed. These results suggest that with our current fMRI protocol, the high‐resolution EPI readout has little effect on our navigator‐based motion and field estimation.

Here, our motion‐corrected reconstruction applied only linear B_0_ correction because of the moderate head motion observed in the volunteers scanned. Although correcting spatially nonlinear B_0_ changes was shown to be effective in reducing image artifacts during instructed head motion,^39^ our prior study^45^ with more subjects (*n* = 11) and longer scan durations (˜35 min) indicated that nonlinear B_0_ correction offers limited benefits in the moderate motion regime, while significantly increasing reconstruction time. Part of our future work is to investigate how applying nonlinear B_0_ correction would improve BOLD fMRI especially when collected with a breathing paradigm for cerebrovascular reactivity mapping.^94^, ^95^

One limitation of the current study is that the MP2RAGE images defining a volunteer's native T1w space were acquired with the RF transmitters operating in its CP mode, thereby compromising tissue segmentation due to nonuniform tissue contrast arising from RF inhomogeneity. Furthermore, due to the use of the BIDS‐compatible sMRIPrep pipeline for structural preprocessing, only T_1_w images could be used to determine tissue segmentation. The tissue segmentation obtained this way is expected to be not as accurate as that attainable using both T_1_w and T_2_w images like in the Human Connectome Project (HCP) structural preprocessing pipeline,^73^ due in large to the inability to differentiate dura from WM. Part of our future work is to follow the HCP structural preprocessing pipeline for improved accuracy in tissue segmentation, for which we will also use the PASTeUR package (https://github.com/FranckMauconduit/MRI‐packages‐siemens/blob/main/PASTeUR‐package/PASTeUR‐package.md) in combination with universal pTx pulses^96^, ^97^, ^98^ designed for our custom‐built RF array at 10.5 T to acquire the required T_1_w MPRAGE^99^ and T_2_w SPACE^100^ images both with improved uniformity of tissue contrast across the brain.

Another limitation of this study is that the volume TR dictating the temporal resolution of BOLD time series was about 2.3 s, longer than that of the 7T HCP fMRI protocol^31^ using 2D simultaneous multislice multiband imaging (which was 1 s) when pursuing nearly identical spatial resolution (1.58 vs. 1.6 mm isotropic for 7T HCP). This was due in large to the fact that we would like to (i) keep the combined acceleration under 15‐fold to prevent excessively large g‐factors, (ii) keep the TE at about 20 ms to retain BOLD contrast, and (iii) avoid partial Fourier to eliminate associated image blurring. These considerations necessitated the collection of two readout segments per *k*_*z*_ plane in the presence of navigator acquisition, giving rise to an over 2‐fold increase in volume TR as compared with the 7T HCP fMRI protocol, in which no navigator is acquired, and a single‐shot readout is achieved per *k*_*z*_ plane through the use of 10‐fold combined acceleration along with 7/8 partial Fourier. Part of our future work is to seek strategies that can be used to shorten the volume TR (thereby improving the temporal resolution), including the use of our custom‐built 128‐channel RF receive array^11^ to enable higher combined acceleration, the use of high‐performance head gradients^101^ to allow shorter echo spacing, and the use of alternative k‐space trajectories^102^, ^103^ to achieve faster navigator acquisition per TR.

Here we showcased the utility of our comprehensive motion‐robust 3D‐GRE‐EPI method in the context of resting‐state fMRI at 10.5 T with spatial resolution nearly the same as in the 7T HCP fMRI protocol. It will be interesting to study how our motion‐robust imaging method would improve resting‐state fMRI at 10.5 T when pursuing higher spatial resolution. It also remains to be investigated how our motion‐robust method can be used to promote task fMRI studies,^20^, ^104^, ^105^ especially those prone to task‐induced motion. It is also of our interest to study how our comprehensive motion‐robust imaging method can be extended to mesoscale fMRI with partial brain coverage.^106^, ^107^, ^108^

As far as correction of rigid‐body head motion itself is concerned, previous work has demonstrated the utility of prospective motion correction^109^ for human‐brain fMRI with a 3D‐EPI sequence, based on real‐time motion tracking using either self‐navigation^110^ or an optical camera^111^. Although shown to be effective in improving image quality at lower field strengths, the efficacy of prospective motion correction at 10.5 T remains unclear. Part of our future work is to investigate how prospective motion correction would compare with our motion‐robust 3D‐EPI method especially when used to collect partial‐brain fMRI data at high spatiotemporal resolution with slab‐selective excitation.

## 5 CONCLUSION

We demonstrated the feasibility of conducting quality whole‐brain BOLD fMRI in humans at 10.5 T by developing a new comprehensive motion‐robust imaging method. Our new method was devised by synergistically combining several leading‐edge techniques including a motion‐robust 3D‐GRE‐EPI sequence (allowing for image reconstruction with navigator‐informed joint motion and B_0_ correction), parallel transmission (allowing for uniform excitation across the brain), and high‐channel‐count RF receive coils (enabling highly accelerated data acquisition). Using a Food and Drug Administration–approved, custom‐built, 16‐channel transmit and 80‐channel receive RF array paired with subject‐specific, parallel‐transmission, water‐selective RF pulse design, we have presented data showing improved quality of fMRI time series, enhancing tSNR while promoting the measurements of resting‐state fMRI metrics including the amplitude of low‐frequency fluctuation and regional homogeneity. This work paves the way for promising future applications at 10.5 T aimed at studying brain function and networks with high spatiotemporal resolution.

## Supporting information


**Figure S1.** Accelerated four‐dimensional (4D) mean images (i.e., averaged in the time dimension) of blood oxygen level–dependent (BOLD) echo‐planar imaging (EPI) time series for reconstruction with motion and field correction (Corrected EPI) versus reconstruction using the same raw data but with no correction (Uncorrected EPI) at both subject and group levels. Shown are 4D mean images for anterior–posterior (AP) phase‐encoding acquisition only, for posterior–anterior (PA) phase‐encoding acquisition only, and for combining both phase‐encoding acquisitions (AP + PA), similar to Figure 1, but now expanded to a full overview of all 5 volunteers scanned.
**Figure S2.** Unprocessed three‐dimensional (3D) echo‐planar imaging (EPI) reconstruction with motion and field correction (Corrected) versus reconstruction using the same raw data but with no correction (Uncorrected). Shown are images of a representative sagittal slice from a single volume of 1 volunteer obtained using anterior–posterior (AP) and posterior–anterior (PA) phase‐encoding directions. Note that for both AP and PA phase‐encoding directions, corrected and uncorrected reconstructions were visually identical to each other, presenting similar image quality with same susceptibility distortion characteristics.
**Figure S3.** The utility of parallel transmission (pTx) for motion‐robust whole‐brain blood oxygen level–dependent (BOLD) functional MRI (fMRI) at 10.5 T. Shown are magnitude images of a single volume in three orthogonal views acquired from a representative volunteer using tailored pTx pulse design versus traditional binomial water excitation in the CP mode (CP). PTx spatial spectral pulses were designed for uniform water excitation across the brain. A custom‐built 16‐transmit (Tx)/80‐receive (Rx) radiofrequency (RF) head array was used for data acquisition. Note how the use of our pTx pulses improved image quality, effectively eliminating RF shading artifacts (*as indicated by arrows*) observed with the CP mode excitation.
**Figure S4.** Temporal derivative of root mean square variance over voxels (DVARS) for reconstruction with motion and field correction (Corrected) versus reconstruction using the same raw data but with no correction (Uncorrected) at the subject level. Shown are time courses of DVARS values (output from the fMRIPrep pipeline) for Corrected (*blue*) versus Uncorrected (*red*) reconstruction. For each volunteer and given reconstruction, the DVARS time course shown was that obtained from the anterior–posterior (AP) phase‐encoding acquisition (the first half of the entire time course), followed by that from the posterior–anterior (PA) phase‐encoding acquisition (the second half of the entire time course). Note that although the two DVARS time courses were highly correlated with each other, the time course for the Corrected reconstruction was different than that for the Uncorrected reconstruction.
**Figure S5.** Comparing functional connectivity for reconstruction with motion and field correction (Corrected) versus reconstruction using the same raw data but with no correction (Uncorrected) at group levels. Shown are the pair‐wise functional connectivity matrices based on Pearson's correlation computed for Corrected (*left column*) versus Uncorrected (*middle column*) reconstruction, along with the mean difference map between the two (*right column*). The 5 volunteers scanned were split into two groups per their framewise displacement (FD) time courses (as shown in Figure 2): the low‐motion group (including Volunteers 1, 2, and 5) and the high‐motion group (including Volunteers 3 and 4). For each group, functional connectivity matrices were derived using the 4S156Parcels atlas for parcellation and based on postprocessed functional MRI (fMRI) time series concatenated across runs and volunteers. The mean difference map, meant to visualize the differences in functional connectivity, was obtained by first taking the difference between the two functional connectivity matrices and then averaging across parcels for each block (representing either intracluster or intercluster functional connectivity). In other words, the value assigned to a block was the mean of the differences calculated as (FC_corrected – FC_uncorrected), where FC_corrected and FC_uncorrected, respectively, refer to parcel‐specific Corrected and Uncorrected functional connectivity values inside that block. Note how image reconstruction with motion and field correction changed the estimation of functional connectivity, especially in the presence of high motion.
**Figure S6.** Temporal signal‐to‐noise ratio (tSNR) maps calculated using unprocessed blood oxygen level–dependent (BOLD) data for reconstruction with motion and field correction (Corrected) versus reconstruction using the same raw data but with no correction (Uncorrected). Shown are tSNR maps in three representative axial slices for Corrected (*leftmost panel*) versus Uncorrected (*middle panel*) reconstruction at subject and group levels, along with associated whole‐brain tSNR histograms (*rightmost panel*). For each volunteer and given reconstruction, the two per‐run tSNR maps were first calculated by considering all the unprocessed 150 image volumes in each run and were averaged to form the final tSNR map. The group tSNR map was created by averaging tSNR maps of each individual across 5 volunteers. In each case, the tSNR histograms of the two reconstructions were created for comparison (where the vertical axis is “Probability,” defined as the number of observations in bin divided by the total number of observations), along with associated mean and max tSNR values reported. Note the tSNR improvement with motion and field correction at both subject and group levels.
**Figure S7.** Temporal signal‐to‐noise ratio (tSNR) maps for reconstruction with field‐only correction (Field‐only corrected). Shown are tSNR maps in three representative axial slices (*left panel*) at the group level, along with the whole‐brain tSNR histogram (*right panel*) in comparison to those obtained for reconstruction using the same raw data but with joint motion and field correction (Corrected), and with no correction (Uncorrected). The group tSNR map was created by averaging tSNR maps of each individual across 5 volunteers. In each case, the tSNR histogram (where the vertical axis is “Probability” defined as the number of observations in bin divided by the total number of observations) was created for comparison, along with associated mean and max tSNR values reported. Note how the field correction improved tSNR relative to the uncorrected case.
**Figure S8.** Root mean square (RMS) of motion estimation from functional MRI (fMRI) preprocessing for reconstruction with motion and field correction (Corrected) versus reconstruction using the same raw data but with no correction (Uncorrected). Shown are RMS values at the group level for six rigid‐body motion parameters (i.e., X, Y, Z translations and pitch, yaw, roll rotations). Motion parameters were estimated on a frame‐by‐frame basis from volume re‐alignment. For each motion parameter, RMS over time was first obtained at the run level and then averaged across all 10 runs (i.e., 5 volunteers each with two runs). The bar in each case indicates the standard deviation of RMS across 10 runs. Note how our Corrected reconstruction effectively reduced the volume misalignment to be addressed in fMRI preprocessing.
**Figure S9.** Examining high‐resolution functional MRI (fMRI) sampling effects on navigator‐based estimation of motion and field parameters. Shown are traces of six rigid‐body motion parameters (X, Y, Z translations and roll, pitch, yaw rotations), three linear field changes (Gx, Gy, Gz), and the global field change (G0), estimated by our motion‐corrected reconstruction using the same navigator collected with high‐resolution echo‐planar imaging (EPI) readout as in our human scan (1.6‐mm anterior–posterior [AP]) versus collected with nearly no EPI readout (No readout). The horizontal axis represents number of repetition times (TRs). Note that the high‐resolution fMRI sampling had little effect on our navigator‐based estimation of motion and field parameters.
**Table S1.** Comparison of correlations of derivative of root mean square variance over voxels (DVARS) and framewise displacement (FD) for our motion‐corrected image reconstruction (Corrected) versus uncorrected image reconstruction (Uncorrected). Reported are the correlations calculated at the run level for Corrected versus Uncorrected reconstruction. For both reconstructions, correlation was quantified using the reconstruction‐specific DVARS time course but using the FD time course from the uncorrected reconstruction, because it closely reflected the intervolume rigid‐body head motion incurred during the blood oxygen level–dependent (BOLD) functional MRI (fMRI) data acquisition. Note that our motion‐corrected reconstruction reduced the correlation of the DVARS and FD time courses in every run and in every volunteer, thanks to its ability to intra‐volume the head‐motion correction.

## Data Availability

The resting‐state BOLD time‐series data along with associated MP2RAGE T1w images and field maps necessary for fMRI preprocessing and postprocessing pipelines can be downloaded at https://openneuro.org/datasets/ds006206/versions/2.0.4. The *MATLAB* code is made publicly available at https://github.com/XiaopingWu2020/ptx‐spsp4d for pTx pulse design, https://github.com/jiaen‐liu/moco for image reconstruction, and https://github.com/ShuxianQU/bids‐pipelines for our custom workflow integrating BIDS pipelines.
