# Supplementary material for: Advancing whole‐brain BOLD functional MRI in humans at 10.5 T with motion‐robust 3D echo‐planar imaging, parallel transmission, and high‐density radiofrequency receive coils
Source: Magn Reson Med. 2025 Oct 17;95(2):1068–88. doi: 10.1002/mrm.70110 (PMC12681312; doi:10.1002/mrm.70110)
Supplement: Supplementary file 1 — Figure S1. Accelerated four‐dimensional (4D) mean images (i.e., averaged in the time dimension) of blood oxygen level–dependent (BOLD) echo‐planar imaging (EPI) time series for reconstruction with motion and field correction (Corrected EPI) versus reconstruction using the same raw data but with no correction (Uncorrected EPI) at both subject and group levels. Shown are 4D mean images for anterior–posterior (AP) phase‐encoding acquisition only, for posterior–anterior (PA) phase‐encoding acquisition only, and for combining both phase‐encoding acquisitions (AP + PA), similar to Figure 1, but now expanded to a full overview of all 5 volunteers scanned. Figure S2. Unprocessed three‐dimensional (3D) echo‐planar imaging (EPI) reconstruction with motion and field correction (Corrected) versus reconstruction using the same raw data but with no correction (Uncorrected). Shown are images of a representative sagittal slice from a single volume of 1 volunteer obtained using anterior–posterior (AP) and posterior–anterior (PA) phase‐encoding directions. Note that for both AP and PA phase‐encoding directions, corrected and uncorrected reconstructions were visually identical to each other, presenting similar image quality with same susceptibility distortion characteristics. Figure S3. The utility of parallel transmission (pTx) for motion‐robust whole‐brain blood oxygen level–dependent (BOLD) functional MRI (fMRI) at 10.5 T. Shown are magnitude images of a single volume in three orthogonal views acquired from a representative volunteer using tailored pTx pulse design versus traditional binomial water excitation in the CP mode (CP). PTx spatial spectral pulses were designed for uniform water excitation across the brain. A custom‐built 16‐transmit (Tx)/80‐receive (Rx) radiofrequency (RF) head array was used for data acquisition. Note how the use of our pTx pulses improved image quality, effectively eliminating RF shading artifacts (as indicated by arrows) observed with the CP mode exci [file MRM-95-1068-s001.pdf]

Supporting Information

Supporting Figures

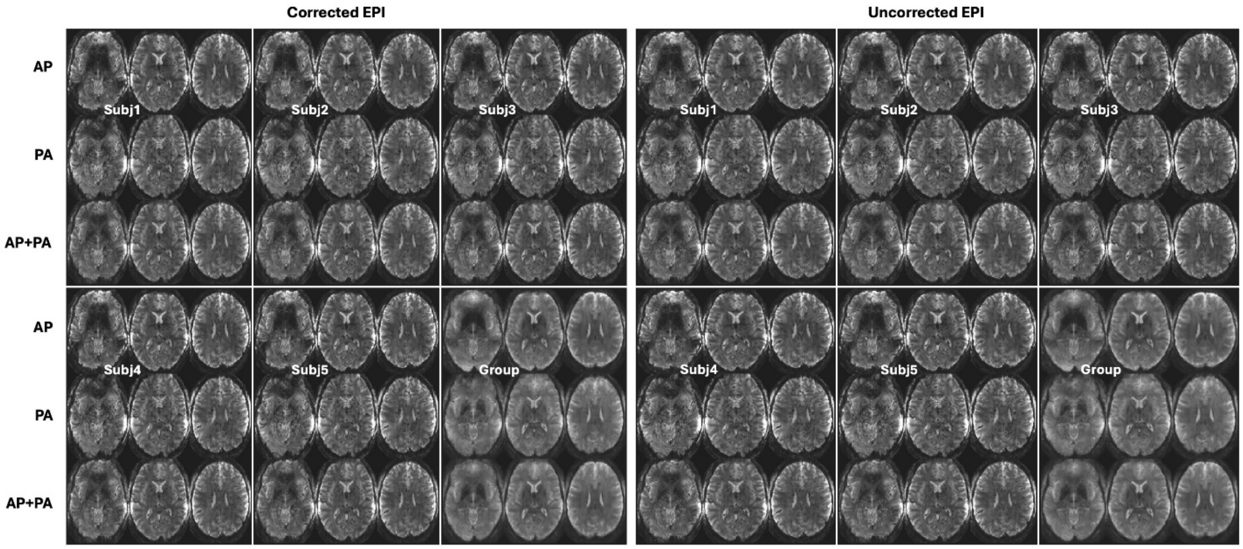

Fig. S1. Accelerated 4D mean images (i.e., averaged in the time dimension) of BOLD EPI timeseries for reconstruction with motion and field correction (Corrected EPI) vs reconstruction using the same raw data but with no correction (Uncorrected EPI) at both subject and group levels. Shown are 4D mean images for Anterior Posterior (AP) phase-encoding acquisition only, for PA phase-encoding acquisition only, and for combining both phase-encoding acquisitions (AP + PA), similar to Fig. 1, but now expanded to a full overview of all five volunteers scanned.

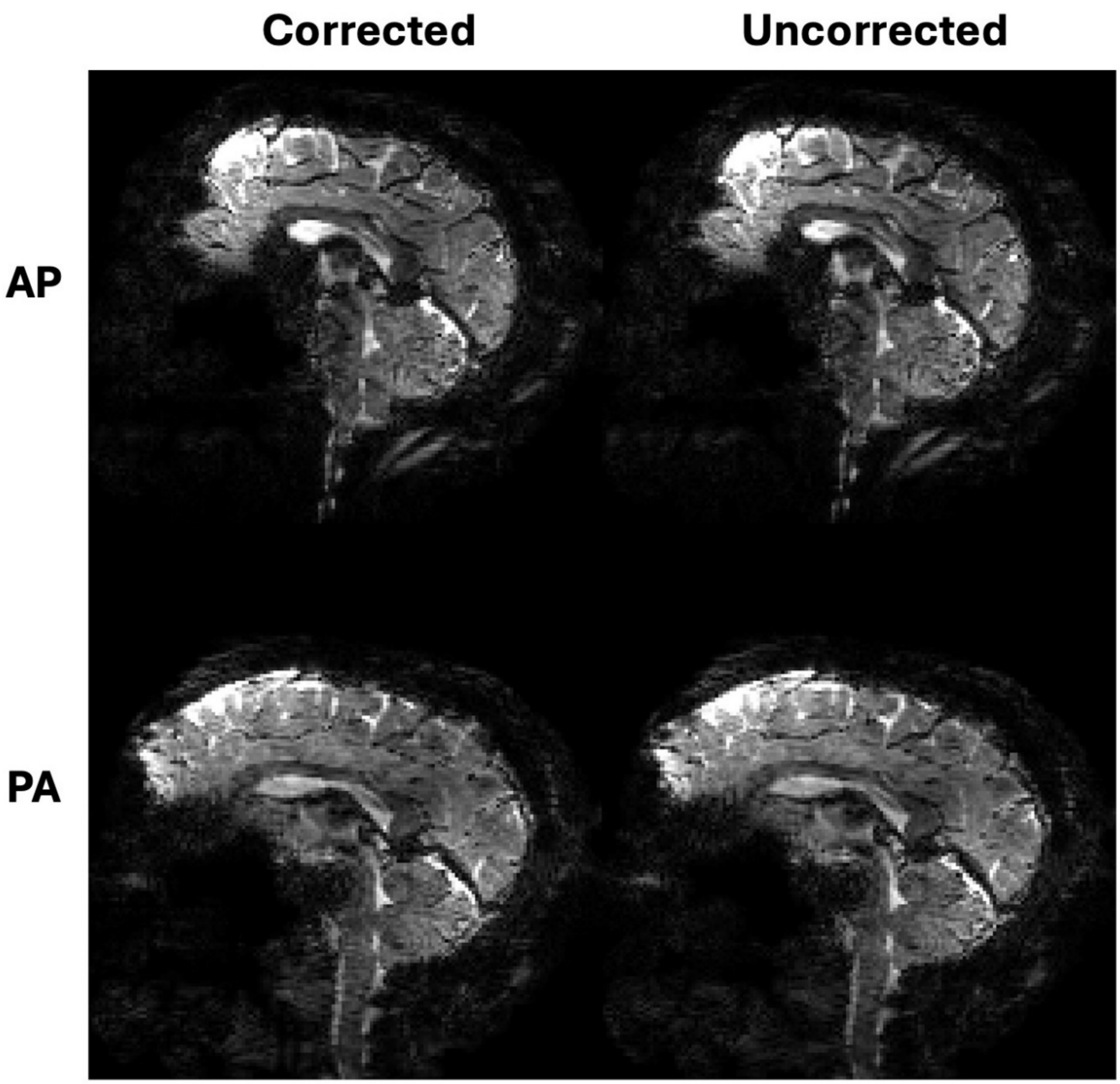

Fig. S2. Unprocessed 3D EPI reconstruction with motion and field correction (Corrected) vs. reconstruction using the same raw data but with no correction (Uncorrected). Shown are images of a representative sagittal slice from a single volume of one volunteer obtained using Anterior-Posterior (AP) and PA phase-encoding directions. Note that for both AP and PA phase-encoding directions, corrected and uncorrected reconstructions were visually identical to each other, presenting similar image quality with same susceptibility distortion characteristics.

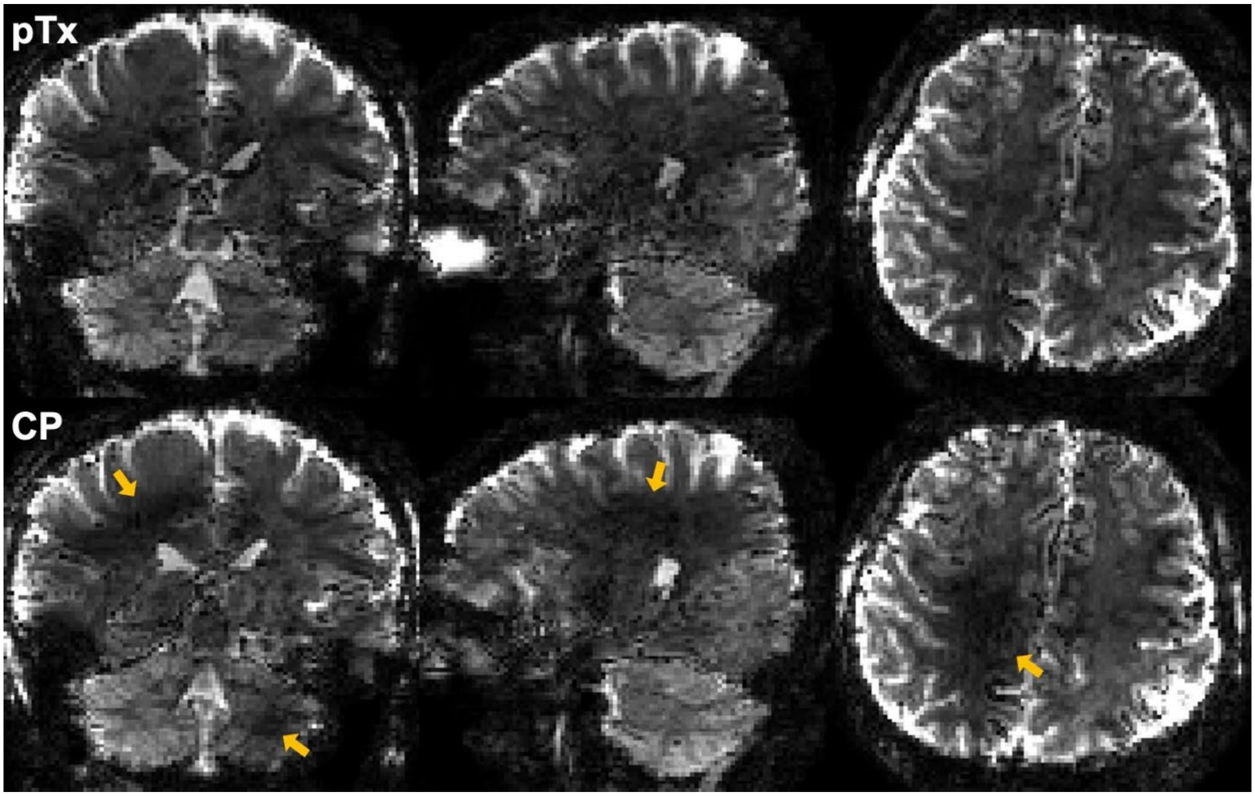

Fig. S3. Demonstrating the utility of parallel transmission (pTx) for motion-robust whole brain BOLD fMRI at 10.5 T. Shown are magnitude images of a single volume in three orthogonal views acquired from a representative volunteer using tailored pTx pulse design (pTx) vs. traditional binomial water excitation in the CP mode (CP). PTx spatial spectral pulses were designed for uniform water excitation across the brain. A custom-built 16Tx80Rx RF head array was used for data acquisition. Note how the use of our pTx pulses improved image quality, effectively eliminating RF shading artifacts (as indicated by arrows) observed with the CP mode excitation.

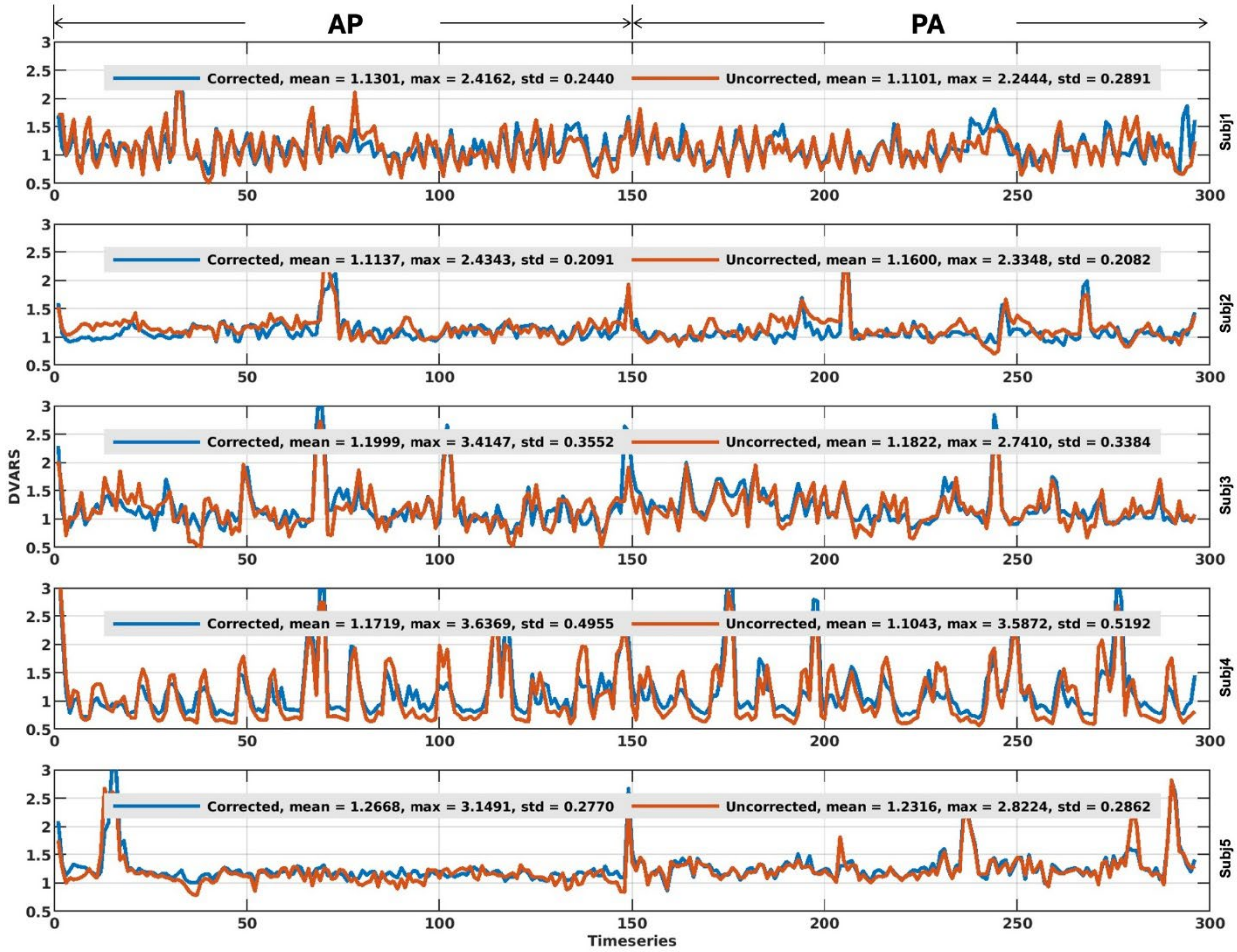

Fig. S4. Temporal derivative of root mean square variance over voxels (DVARS) for reconstruction with motion and field correction (Corrected) vs. reconstruction using the same raw data but with no correction (Uncorrected) at the subject level. Shown are time courses of DVARS values (output from the fMRIPrep pipeline) for Corrected (blue) vs. Uncorrected (red) reconstruction. For each volunteer and given reconstruction, the DVARS time course shown was that obtained from the AP phase encoding acquisition (the first half of the entire time course), followed by that from the PA phase encoding acquisition (the second half of the entire time course). Note that although the two DVARS time courses were highly correlated with each other, the time course for the Corrected reconstruction was different than that for the Uncorrected reconstruction.

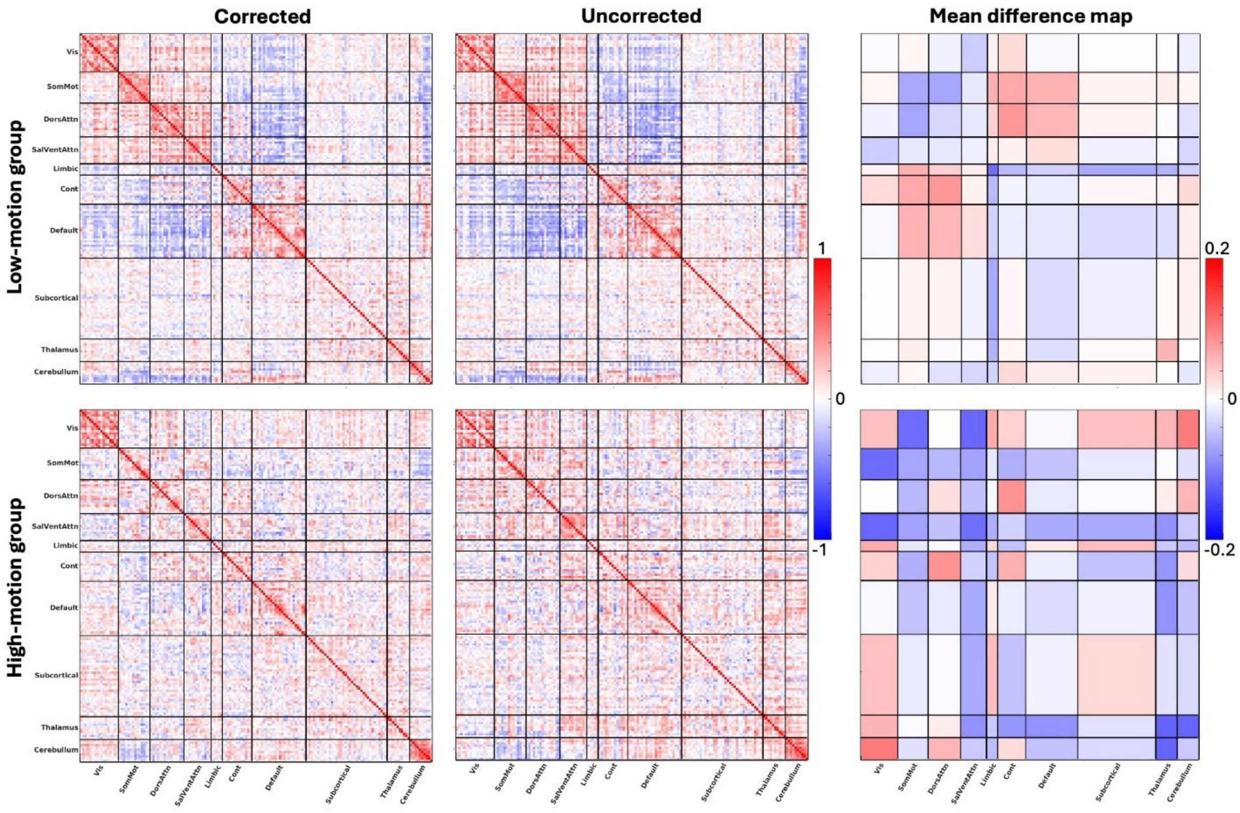

Fig. S5. Comparing functional connectivity for reconstruction with motion and field correction (Corrected) vs. reconstruction using the same raw data but with no correction (Uncorrected) at group levels. Shown are the pair-wise functional connectivity matrices based on Pearson's correlation computed for Corrected (left column) vs. Uncorrected (middle column) reconstruction, along with the mean difference map between the two (right column). The five volunteers scanned were split into two groups per their FD time courses (as shown in Fig. 2): the low-motion group (including volunteers #1, #2, and #5) and the high-motion group (including volunteers #3, and #4). For each group, functional connectivity matrices were derived using the 4S156Parcels atlas for parcellation and based on postprocessed fMRI timeseries concatenated across runs and volunteers. The mean difference map, meant to visualize the differences in functional connectivity, was obtained by first taking the difference between the two functional connectivity matrices and then averaging across parcels for each block (representing either intra- or inter-cluster functional connectivity). In other words, the value assigned to a block was the mean of the differences calculated as (FC_corrected - FC_uncorrected) where FC_corrected and FC_uncorrected, respectively, refer to parcel-specific Corrected and Uncorrected functional connectivity values

inside that block. Note how image reconstruction with motion and field correction changed the estimation of functional connectivity, especially in the presence of high motion.

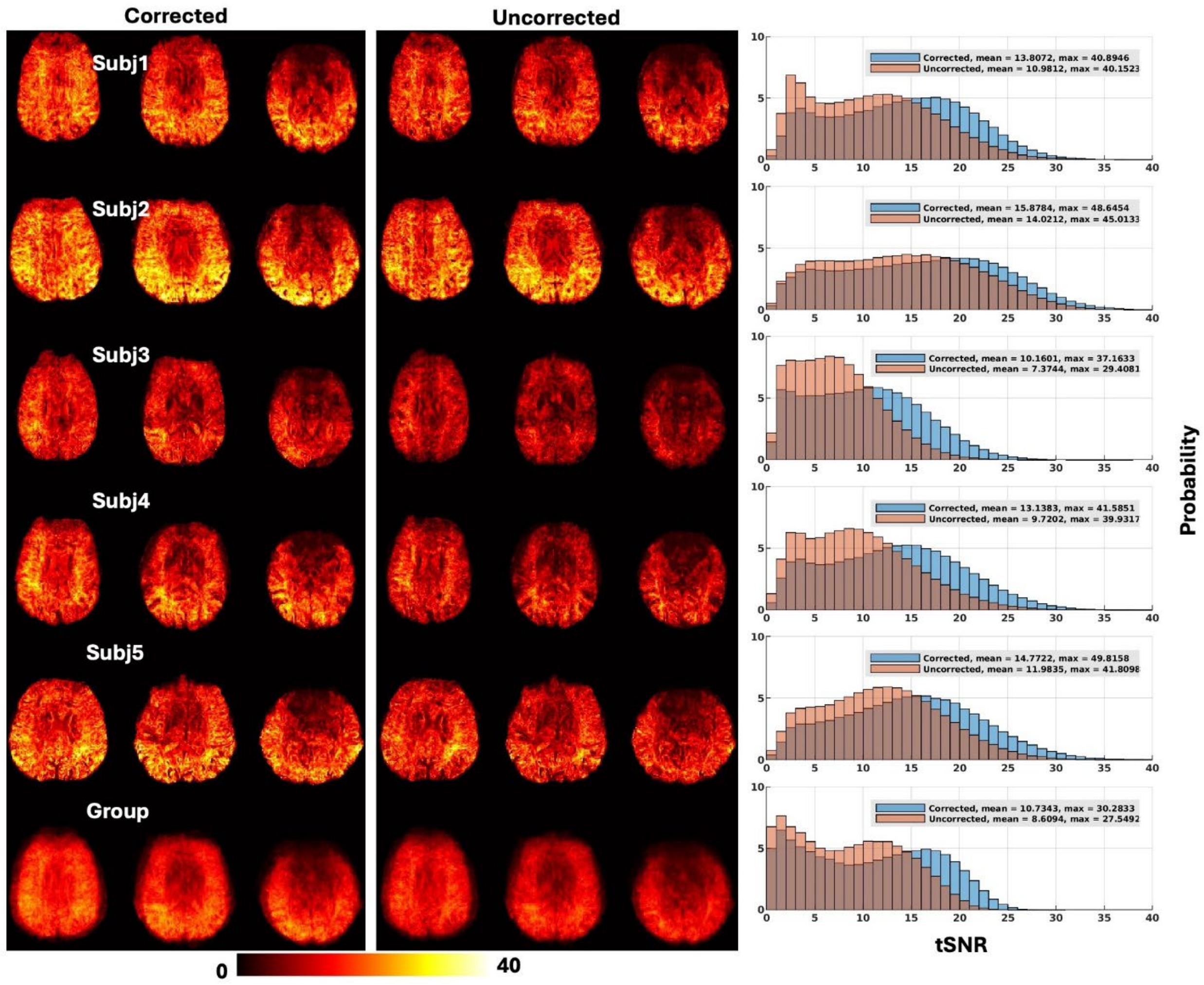

Fig. S6. Temporal SNR maps calculated using unprocessed BOLD data, for reconstruction with motion and field correction (Corrected) vs. reconstruction using the same raw data but with no correction (Uncorrected). Shown are tSNR maps in three representative axial slices for Corrected (leftmost panel) vs. Uncorrected (middle panel) reconstruction at subject and group levels, along with associated whole-brain tSNR histograms (rightmost panel). For each volunteer and given reconstruction, the two per run tSNR maps were first calculated by considering all the unprocessed 150 image volumes in each run, and were averaged to form the final tSNR map. The group tSNR map was created by averaging tSNR maps of each individual across five volunteers. In each case,

the tSNR histograms of the two reconstructions were created for comparison (where the vertical axis is "Probability" defined as the number of observations in bin divided by the total number of observations), along with associated mean and max tSNR values reported. Note tSNR improvement with motion and field correction at both subject and group levels.

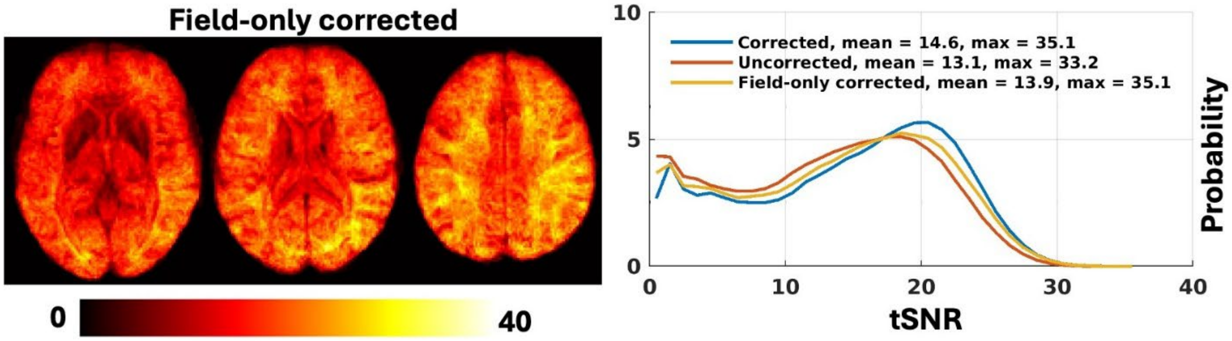

Fig. S7. Temporal SNR maps for reconstruction with field-only correction (Field-only corrected). Shown are tSNR maps in three representative axial slices (left panel) at the group level, along with the whole-brain tSNR histogram (right panel) in comparison to those obtained for reconstruction using the same raw data but with joint motion and field correction (Corrected), and with no correction (Uncorrected). The group tSNR map was created by averaging tSNR maps of each individual across five volunteers. In each case, the tSNR histogram (where the vertical axis is "Probability" defined as the number of observations in bin divided by the total number of observations) was created for comparison, along with associated mean and max tSNR values reported. Note how field correction improved tSNR relative to the uncorrected case.

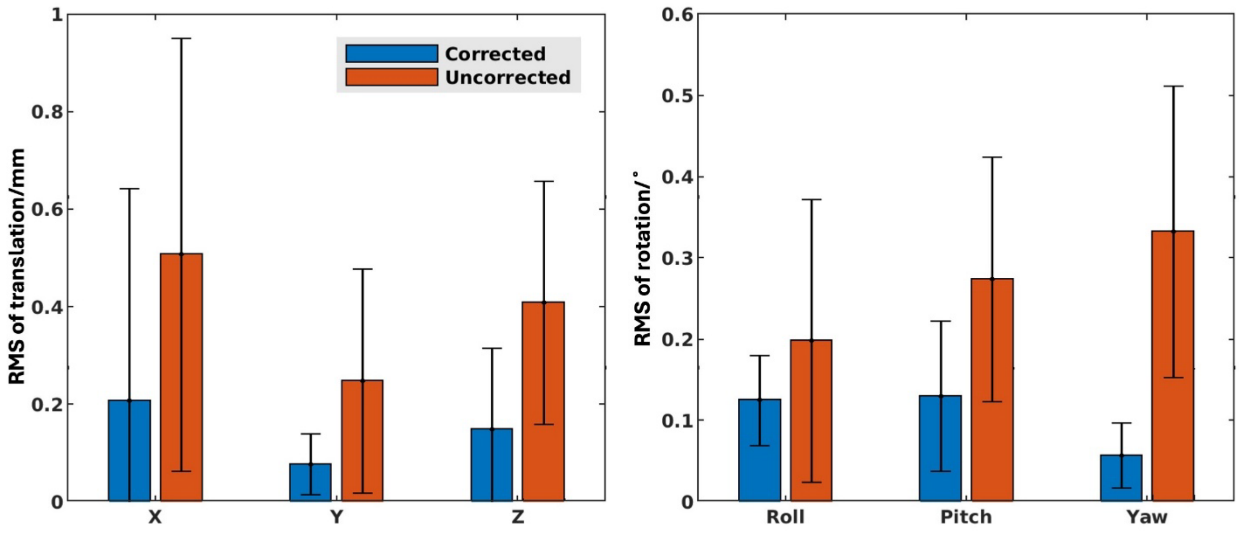

Fig. S8. Root mean square (RMS) of motion estimation from fMRI preprocessing for reconstruction with motion and field correction (Corrected) vs. reconstruction using the same raw data but with no correction (Uncorrected). Shown are RMS values at the group level for six rigid-body motion parameters (i.e., X, Y, Z translations and pitch, yaw, roll rotations). Motion parameters were estimated on a frame-by-frame basis from volume re-alignment. For each motion parameter, RMS over time was first obtained at the run level and then averaged across all 10 runs (i.e., five volunteers each with two runs). The bar in each case indicates the standard deviation of RMS across 10 runs. Note how our Corrected reconstruction effectively reduced the volume misalignment to be addressed in fMRI preprocessing.

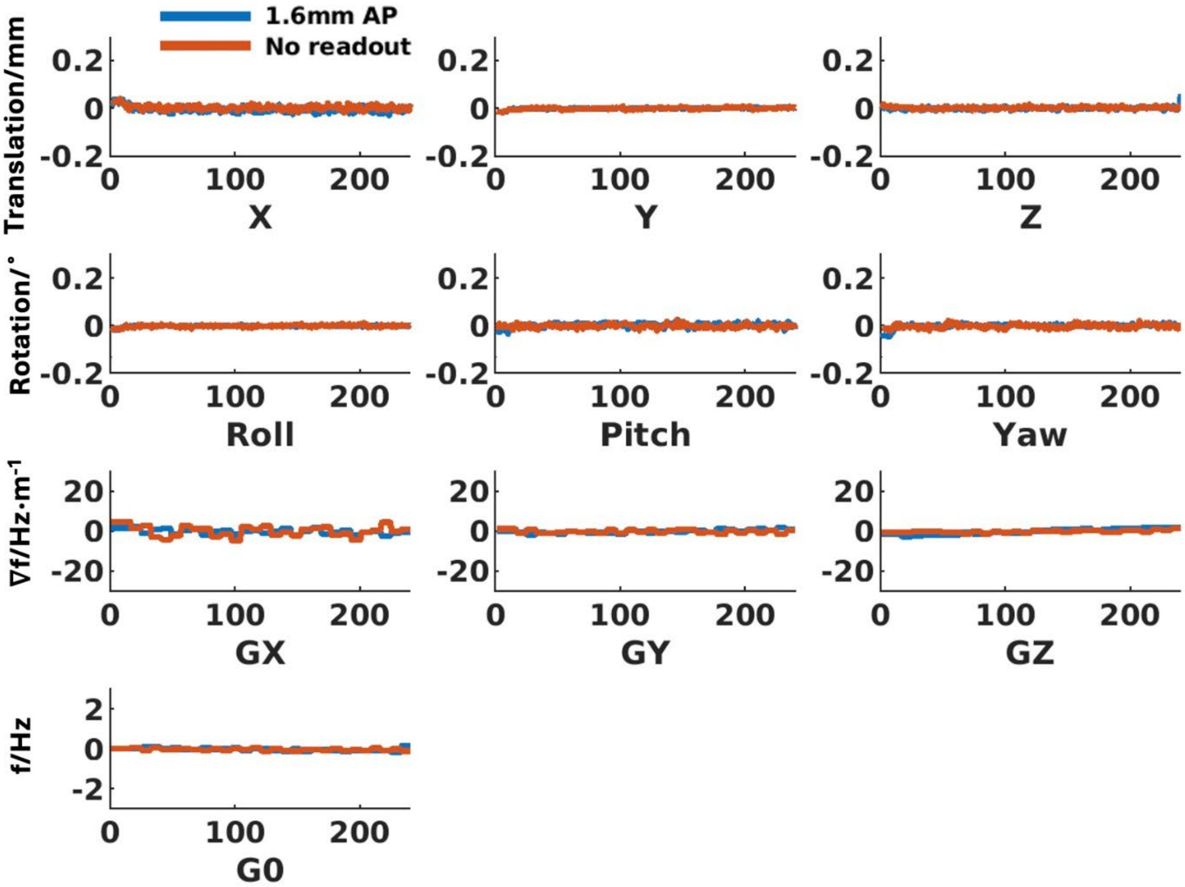

Fig. S9. Examining high resolution fMRI sampling effects on navigator-based estimation of motion and field parameters. Shown are traces of six rigid-body motion parameters (X, Y, Z translations and roll, pitch, yaw rotations), three linear field changes (Gx, Gy, Gz), and the global field change (G0), estimated by our motion-corrected reconstruction using the same navigator collected with high resolution EPI readout as in our human scan (1.6 mm AP) vs. collected with nearly no EPI readout (No readout). The horizontal axis represents number of TRs. Note that the high resolution fMRI sampling had little effects on our navigator based estimation of motion and field parameters.

Supporting Tables

	Subj1		Subj2		Subj3		Subj4		Subj5	
	AP	PA	AP	PA	AP	PA	AP	PA	AP	PA
Corrected	0.1789	0.2265	0.2124	0.2812	0.5424	0.2613	0.3563	0.1744	0.1656	0.8246
Uncorrected	0.3465	0.3544	0.3228	0.4104	0.6546	0.4445	0.6223	0.5045	0.3614	0.8869

Tab. S1. Comparison of correlations of DVARS and FD for our motion-corrected image reconstruction (Corrected) vs. uncorrected image reconstruction (Uncorrected). Reported are the correlations calculated at the run level for Corrected vs. Uncorrected reconstruction. For both reconstructions, correlation was quantified using the reconstruction-specific DVARS time course, but using the FD time course from the uncorrected reconstruction since it closely reflected the inter-volume rigid-body head motion incurred during the BOLD fMRI data acquisition. Note that our motion-corrected reconstruction reduced the correlation of the DVARS and FD time courses in every run and in every volunteer thanks to its ability to intra-volume head motion correction.
